# A Process-Based Model of TCA Cycle Functioning to Analyze Citrate Accumulation in Pre- and Post-Harvest Fruits

**DOI:** 10.1371/journal.pone.0126777

**Published:** 2015-06-04

**Authors:** Audrey Etienne, Michel Génard, Christophe Bugaud

**Affiliations:** 1 UMR QUALISUD, Centre de Coopération International en Recherche Agronomique pour le Développement (CIRAD), Campus Agro-Environnemental Caraïbe, Lamentin, France; 2 UR 1115 Plantes et Systèmes de Cultures Horticoles, INRA, Avignon, France; 3 UMR QUALISUD, CIRAD, Montpellier, France; National University of Rosario, ARGENTINA

## Abstract

Citrate is one of the most important organic acids in many fruits and its concentration plays a critical role in organoleptic properties. The regulation of citrate accumulation throughout fruit development, and the origins of the phenotypic variability of the citrate concentration within fruit species remain to be clarified. In the present study, we developed a process-based model of citrate accumulation based on a simplified representation of the TCA cycle to predict citrate concentration in fruit pulp during the pre- and post-harvest stages. Banana fruit was taken as a reference because it has the particularity of having post-harvest ripening, during which citrate concentration undergoes substantial changes. The model was calibrated and validated on the two stages, using data sets from three contrasting cultivars in terms of citrate accumulation, and incorporated different fruit load, potassium supply, and harvest dates. The model predicted the pre and post-harvest dynamics of citrate concentration with fairly good accuracy for the three cultivars. The model suggested major differences in TCA cycle functioning among cultivars during post-harvest ripening of banana, and pointed to a potential role for NAD-malic enzyme and mitochondrial malate carriers in the genotypic variability of citrate concentration. The sensitivity of citrate accumulation to growth parameters and temperature differed among cultivars during post-harvest ripening. Finally, the model can be used as a conceptual basis to study citrate accumulation in fleshy fruits and may be a powerful tool to improve our understanding of fruit acidity.

## Introduction

Citrate is one of the most important organic acids in many fruits [1], and its concentration in the pulp plays a critical role in organoleptic properties [2–5]. The citrate concentration varies considerably among cultivars of many fruit species including citrus [6], peach [7], pineapple [8], and banana [5]. The accumulation of citrate in fruit cells is a complex phenomenon because it involves several metabolic pathways and transport mechanisms across different compartments, mainly cytosol, mitochondria, and vacuole (for review see [9]). Ongoing transcriptomic [10], metabolomic [11], proteomic [12], and QTL studies [13] have begun to elucidate the complexity of the mechanisms involved in citrate accumulation. However, the regulation of citrate accumulation throughout fruit development, and the origins of the phenotypic variability of the citrate concentration within fruit species remain to be clarified. Given the complexity of the processes involved, ecophysiological process-based simulation models (PBSMs) could advance our understanding of the physiological mechanisms underlying citrate accumulation [14]. PBSMs could also help to elucidate the differences in citrate accumulation among and within fruit species, as it is the case for sugar accumulation in peach [15], and grape berry [16].

Attempts to mechanistically model citrate accumulation in fruits are rare. Lobit et al. [17] proposed a mechanistic model to simulate the dynamics of citrate concentration in peach fruit. This model was based on the assumption that in fleshy fruits, citrate accumulation is driven by the TCA cycle located in the mitochondria, which is a convincing hypothesis [9]. This model was used to analyze the effects of temperature and pulp growth on citrate concentrations in two cultivars of peach [17, 18], and appeared to provide a good framework to study citrate accumulation in fleshy fruit. However, the approach used to solve the system of equations derived from the model led to a simple equation to predict the rate of net citrate production with parameters that had lost their real biological meaning. Thus, despite its good predictive quality, this model did not allow a complete study of the behavior and regulation of fruit mitochondrial citrate metabolism.

In the present study, we built a new, more mechanistic, model of citrate accumulation, with parameters that have biological meaning, based on a simplified representation of the TCA cycle adopted by Lobit et al. [17], to predict citrate concentration in fruit during the pre- and post-harvest stages. Banana fruit was taken as a reference because it has the particularity of having separate growth and post-harvest ripening stages, during which citrate concentration undergoes substantial changes [19]. Moreover, the concentration of citrate in banana pulp varies greatly among cultivars which make possible to use the model as a tool to analyze the genotypic variability [5, 20]. The physiological age of the fruit at harvest is known to affect the concentration of citrate in banana pulp during post-harvest ripening [21]. Fruit pruning and potassium fertilization, two cultural practices commonly used by banana growers, can also impact the concentration of citrate in fleshy fruits (for review see [9]). Consequently, we chose to calibrate and validate the model on three cultivars with contrasting citrate accumulation, grown under different fruit loads and potassium supplies, and harvested at different stages to study how these growing conditions affect citrate metabolism (or not). Model parameterization, model selection, and test of fit are presented for the pre- and post-harvest phases. The sensitivity of the model to the input variables was analyzed during the pre- and post-harvest stages. The model enabled us to (i) advance our understanding of citrate metabolism during growth and post-harvest ripening of banana fruit; (ii) propose a possible explanation for differences in citrate accumulation among cultivars and identify potential genotypic parameters (i.e. genotype-dependent parameters); and (iii) study the effects of fruit growth conditions on citrate metabolism. Finally, the model can be used as a conceptual basis to study citrate accumulation in pre- and post-harvest stages in fleshy fruits and thus may be a powerful tool to improve our understanding of fruit acidity.

## Materials and Methods

Description and units of parameters, constants and variables used throughout the text are summarized in S1 Table.

### Model description

#### Model hypothesis

**Fig 1:**
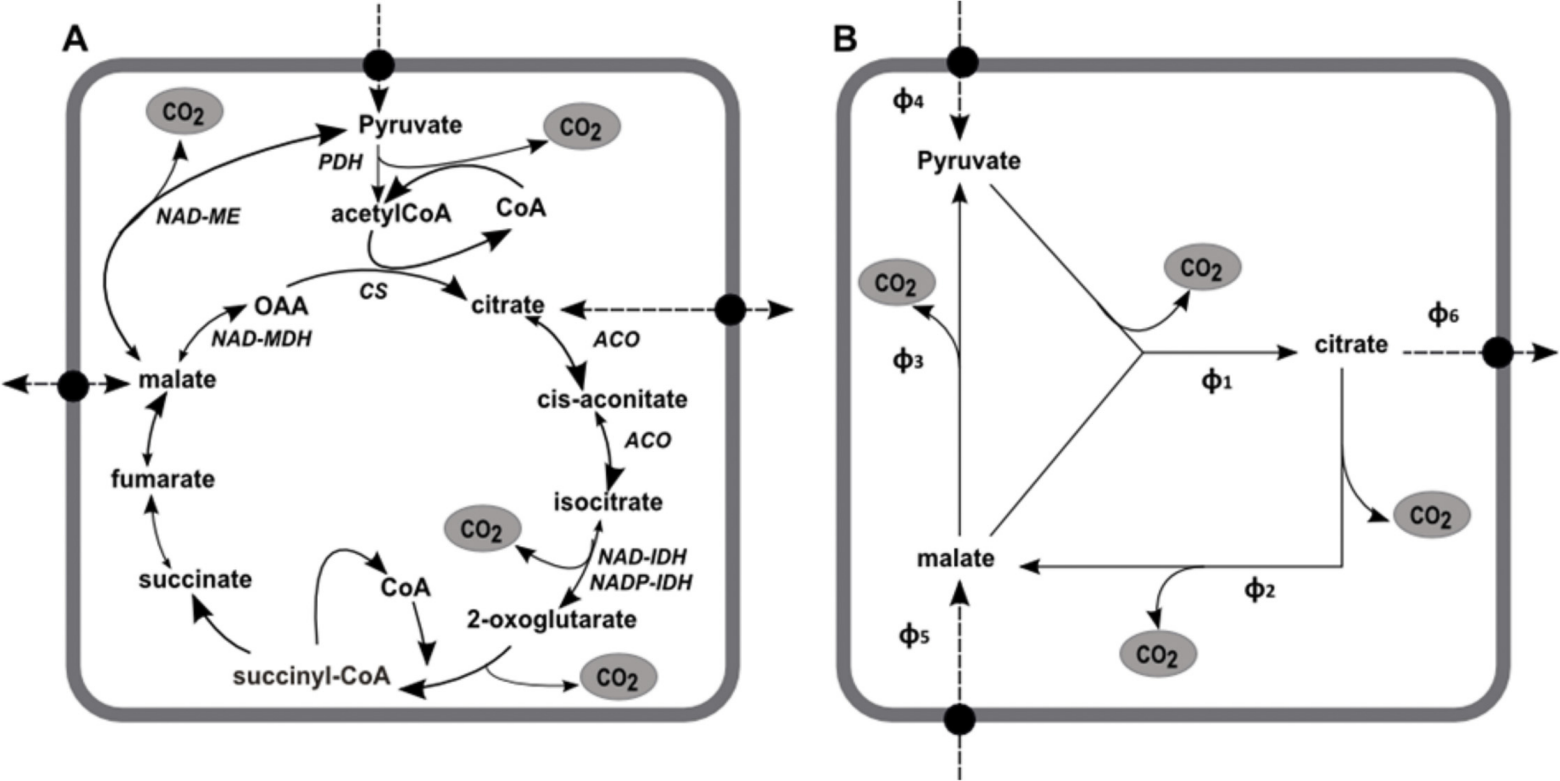
Schematic representation of the TCA cycle. (A) Reactions of the TCA cycle in the mitochondria and (B) the simplified model of Lobit et al. (2003). Enzymes are in italics: ACO, aconitase; CS: citrate synthase; NAD-ME, NAD-malic enzyme; NAD-IDH, NAD-isocitrate dehydrogenase; NADP-IDH, NADP- isocitrate dehydrogenase; NAD-MDH, NAD-malate dehydrogenase; PDH, pyruvate dehydrogenase. Dashed arrows indicate transport across the mitochondrial membrane.

The accumulation of citrate in fruit cells involves several metabolic pathways and transport mechanisms across different compartments, mainly cytosol, mitochondria, and vacuole. Nevertheless, we showed in a previous paper [9] that the accumulation of citrate in the vacuole is unlikely to be limited by thermodynamic conditions, on the contrary to malate [22]. Therefore, it is likely that citrate accumulation in the vacuole is controlled by its cytosolic concentration and consequently by its metabolism. Among several possible pathways related to citrate metabolism, the TCA cycle is the only one that allows citrate synthesis. Therefore, the present model is based on the assumption that citrate accumulation in fleshy fruit is driven by the TCA cycle located in the mitochondria. The TCA cycle results in the oxidation of pyruvate into CO_2_ and a reduction in co-enzymes through a series of conversions between organic acids including malate and citrate (Fig 1A). The maintenance of the pools of TCA cycle intermediates implies that for each metabolite exported, one is imported, and vice versa. These exchanges are achieved by a variety of mechanisms mediated by mitochondrial carrier proteins (for reviews, see [23, 24]). The model presented here is based on the simplified representation of the TCA cycle used in the model of Lobit et al. [17] (Fig 1B). The only metabolites considered, pyruvate, malate and citrate, were chosen because they are at branch points between several reactions and because they are exchanged between the cytosol and the mitochondria. Pulp fruit was considered as a single big cell with mitochondrial and cytosolic compartments. We did not consider the compartmentalization of citrate in the vacuole because as we mentioned above we assumed that this step is not limiting for citrate accumulation in fruit cells.

#### Stoichiometric equations

The maintenance of the pools of metabolic intermediates imposes, for any given metabolite, that the sum of metabolic fluxes that synthesizes it and the sum of metabolic fluxes that degrades it are equal (Fig 1B).

$$\frac{dMpyr_{mt}}{dt}=\varphi_{4}+\varphi_{3}-\varphi_{1}=0$$ (1)

$$\frac{dMmal_{mt}}{dt}=\varphi_{5}+\varphi_{2}-\varphi_{3}-\varphi_{1}=0$$ (2)

$$\frac{dMcit_{mt}}{dt}=\varphi_{1}-\varphi_{2}-\varphi_{6}=0$$ (3)

The respiratory flux, approximated as the flux of CO_2_ produced by the TCA cycle, is:

$$Resp=\varphi_{1}+2\varphi_{2}+\varphi_{3}$$ (4)

where Mpyr_mt_, Mmal_mt_, and Mcit_mt_ (mmol) are respectively the amount of pyruvate, malate and citrate in the pulp mitochondrial compartment, φ_i_ are the metabolic fluxes of the TCA cycle (mmol day^-1^), and Resp is the respiratory flux of the pulp mitochondrial compartment (mmol day^-1^).

#### Mathematical representations of enzymatic reactions

The metabolic flux between two compounds is described by enzyme kinetic rate laws, as a function that depends on the concentration of the reactants and on the k_i_ parameters called rate constants [25].

$$\varphi_{1}=k_{1}Cmal_{mt}Cpyr_{mt}$$ (5)

$$\varphi_{2}=k_{2}Ccit_{mt}$$ (6)

$$\varphi_{3}=k_{3}Cmal_{mt}$$ (7)

where k_1_ (L^2^ day^-1^ mmol^-1^), k_2_ (L day^-1^), k_3_ (L day^-1^) are the rate constants, and Cmal_mt_, Cpyr_mt_, Ccit_mt_ are the concentrations of malate, pyruvate and citrate in the pulp mitochondrial compartment, respectively (mmol L^-1^).

#### Mathematical representations of transport reactions

Several carriers are present on the inner membrane of plant cell mitochondria and allow the exchange of metabolites of the TCA cycle between the cytosol and the mitochondrial matrix (for review see [24]). For the sake of simplicity, we assumed that the transport of citrate, malate and pyruvate across the mitochondria depends mainly on the concentration gradients of the species transported between the cytosol and the mitochondrial matrix. Therefore, the formalism adopted to model the transport reactions was derived from Fick’s law, which states that the diffusion flux of a compound is proportional to the concentration gradient of this compound across the membrane.

$$\varphi_{4}=K_{4}(Cpyr_{cyt}-Cpyr_{mt})$$ (8)

$$\varphi_{5}=K_{5}(Cmal_{cyt}-Cmal_{mt})$$ (9)

$$\varphi_{6}=K_{6}(Ccit_{mt}-Ccit_{cyt})$$ (10)

where K_4_, K_5_, K_6_ are membrane permeability (L day^-1^); and Cmal_cyt_, Cpyr_cyt_, Ccit_cyt_ are the respective concentrations of malate, pyruvate and citrate in the pulp cytosolic compartment (mmol L^-1^).

#### Solving the system and expressing the rate of net citrate production φ_6_ (mmol day^-1^)

Replacing the expressions of the different metabolic fluxes in Eq 1 to 4 gives the following system:

$$K_{4}Cpyr_{cyt}-K_{4}Cpyr_{mt}+k_{3}Cmal_{mt}-k_{1}Cmal_{mt}Cpyr_{mt}=0$$ (11)

$$K_{5}Cmal_{cyt}+k_{2}Ccit_{mt}-(K_{5}+k_{3})Cmal_{mt}-k_{1}Cmal_{mt}Cpyr_{mt}=0$$ (12)

$$K_{6}Ccit_{cyt}+k_{1}Cmal_{mt}Cpyr_{mt}-(k_{2}+K_{6})Ccit_{mt}=0$$ (13)

$$2k_{2}Ccit_{mt}+k_{3}Cmal_{mt}+k_{1}Cmal_{mt}Cpyr_{mt}=Resp$$ (14)

where Cpyr_mt_, Cmal_mt_, Ccit_mt_, Ccit_cyt_ are the unknowns of the system, and K_i_, k_i_, Cpyr_cyt_, Cmal_cyt_ are parameters.

The system was solved by using the software Maple (Maple (16). Maplesoft, a division of Waterloo Maple Inc., Waterloo, Ontario). The solutions of Ccit_mt_ and Ccit_cyt_ were put into Eq 10 which gave the following expression for φ_6_:

$$\varphi_{6}=-\frac{1}{4}\frac{\left(k_{3}+K_{5}\right)Resp}{3k_{3}+K_{5}}+\frac{1}{4}\frac{1}{k_{1}\left(3k_{3}+K_{5}\right)}(3K_{4}k_{1}\left(k_{3}+K_{5}\right)Cpyr_{cyt}+K_{5}k_{1}\left(2K_{5}+10k_{3}\right)Cmal_{cyt}-(k_{3}+K_{5}){\left(18K_{4}^{2}Cpyr_{cyt}k_{1}k_{3}+12K_{4}^{2}Cpyr_{cyt}k_{1}K_{5}+8K_{4}K_{5}^{2}Cmal_{cyt}k_{1}+9K_{4}^{2}k_{3}^{2}+4K_{4}^{2}K_{5}^{2}+9K_{4}^{2}Cpyr_{cyt}^{2}k_{1}^{2}+12K_{4}^{2}k_{3}K_{5}+4K_{5}^{2}Cmal_{cyt}^{2}k_{1}^{2}-12K_{4}Cpyr_{cyt}k_{1}^{2}K_{5}Cmal_{cyt}+36K_{4}k_{3}K_{5}Cmal_{cyt}k_{1}+\left(18K_{4}k_{3}k_{1}-6K_{4}Cpyr_{cyt}k_{1}^{2}+4K_{4}K_{5}k_{1}+4K_{5}Cmal_{cyt}k_{1}^{2}\right)Resp+Resp^{2}k_{1}^{2}\right)}^{\frac{1}{2}}+K_{4}\left(5k_{3}K_{5}+3k_{3}^{2}+2K_{5}^{2}\right))$$ (15)

#### Pulp respiration

Pulp respiration during fruit growth was calculated using the growth-maintenance equation [26]. Growth respiration is considered to be proportional to the fruit growth rate and maintenance respiration to dry mass and temperature [27, 28]. The effect of temperature is described with the Q_10_ concept. Pulp respiration (mmol CO_2_ day^-1^) was calculated as the sum of growth and maintenance respiration:

$$Resp=q_{g}\frac{dDW}{dt}+q_{m}DWQ_{10}^{\frac{\theta -20}{10}}$$ (16)

where q_g_ is the growth respiration coefficient (mmol CO_2_ g^-1^), q_m_ is the maintenance coefficient at 20°C (mmol CO_2_ g^-1^ day^-1^), Q_10_ is the temperature ratio of maintenance respiration (dimensionless), DW is the pulp dry weight (g) and Ѳ is the temperature (°C).

Pulp respiration during post-harvest ripening was calculated by considering growth respiration equal to zero, since the fruit was detached.

#### Calculation of the concentration of citrate in the pulp

Citrate concentration in the fruit was obtained by integrating φ_6_ over the monitored period starting with citrate content observed at the beginning of the period, and by dividing it by the pulp fresh weight.

$$Ccit_{t}=\frac{100}{FW_{t}}*(Mcit_{t_{0}}+\int_{t_{0}}^{t}\varphi_{6}dt)$$ (17)

where Ccit (mmol 100g FW^-1^) is the citrate concentration in the pulp, Mcit_to_ (mmol fruit^-1^) is the amount of citrate in the pulp at t_0_, FW is pulp fresh weight (g), t is the time (days after bloom or days after ethylene treatment), t_o_ is the time of the beginning of the experiment, and φ_6_ the rate of net citrate production (mmol day^-1^).

#### Rate constants (k_i_) and membrane permeability (K_i_)

**Fig 2:**
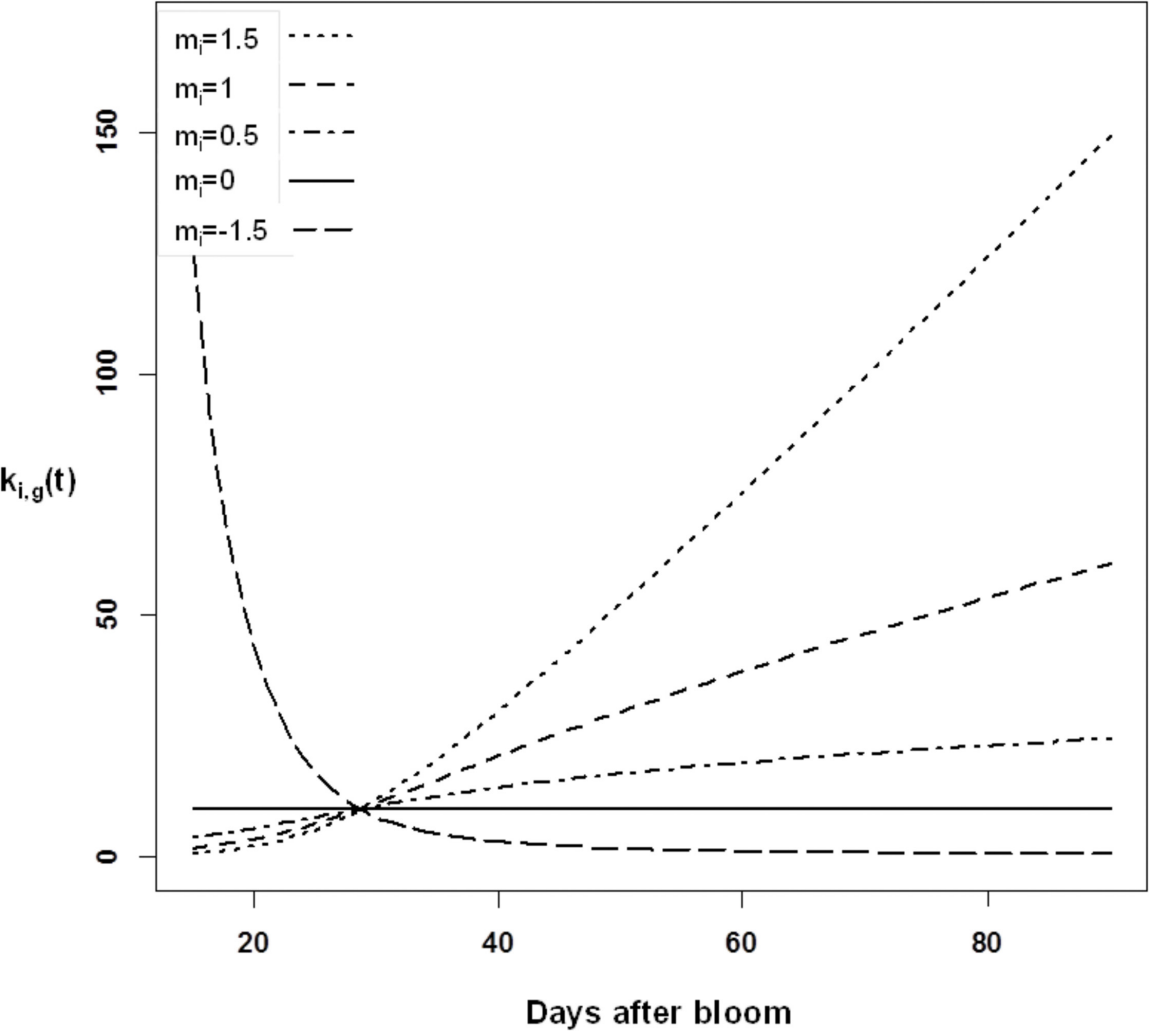
Hypothetical changes in the rate constant k_i,g_(t) during fruit growth as a function of parameter m_i_. k_**i,g**_(t) = *k*_*i*,g_*SDW^*mi*^, with *k*_***i*,*g***_ arbitrarily equal to 10 and SDW (pulp structural dry weight) taking values of the PL cultivar. The different values of m_**i**_ correspond to the following situations: m_**i**_<0: enzyme inhibition exceeds the increase in the number of mitochondria; m_**i**_ = 0: enzyme inhibition compensates for the increase in the number of mitochondria; 0<m_**i**_<1: enzyme inhibition under-compensates for the increase in the number of mitochondria; m_**i**_>1: activation of enzyme along with the increase in the number of mitochondria.

We hypothesized that k_i_ depends on enzyme activity, and K_i_ on transporter activity during fruit development. The activities of the TCA cycle enzymes and of the mitochondrial organic acid transporters can vary or remain constant during fruit growth [11, 29–31], and postharvest ripening [32–34], suggesting that k_i_ and K_i_ are likely to vary during fruit growth and post-harvest ripening. During fruit growth, variations in k_i_ and K_i_ may be due to changes in the number of mitochondria on one hand, and to the regulation of enzymes and transporter activities on the other hand. The first source of variation, i.e. the number of mitochondria, is likely to increase during fruit growth due to cell division and enlargement. Indeed, Winter et al. (1993 and 1994) found a positive linear relationship between the section area of the mitochondrial compartment and the section area of the leaf cells. Therefore, we chose to symbolize the link between the number of pulp mitochondria, and k_i_ and K_i_ by representing their variations during fruit growth as a function of the structural dry weight of the pulp, which represents the constitutive part of pulp cells and is therefore an indicator of pulp cell growth. Concerning the second source of variation, it is known that mitochondrial enzymes and transporters can be regulated by allosteric and post translational regulation, but little information is available on this subject (for review see [35]). Therefore, for the sake of simplicity, we included a regulatory factor (m_i_) to modulate the relationship between the k_i_ and K_i_, and the structural dry weight of the pulp:

$$k_{i,g}(t)=k_{i,g}*{\left(\frac{SDW(t)}{SDW_{ref}}\right)}^{m_{i}}$$ (18)

$$K_{i,g}(t)=K_{i,g}*{\left(\frac{SDW(t)}{SDW_{ref}}\right)}^{m_{i}}$$ (19)

where SDW is the structural dry weight of the pulp (g); SDW_ref_ is a reference structural dry weight equal to 1 g; *k*_*i*,*g*_ (L day^-1^ or L^2^ day^-1^ mmol^-1^), *K*_*i*,*g*_ (L day^-1^), and *m*_*i*_ (dimensionless) are fixed parameters, with *k*_*i*,*g*_ and *K*_*i*,*g*_ positives, and *m*_*i*_ positive, null or negative. Depending on the values of the parameter *m*_*i*_, the patterns of k_i,g_(t) and K_i,g_(t) can remain constant, increase, or decrease, as shown in Fig 2.

During postharvest ripening, the number of mitochondria of the pulp is likely to vary little since the fruit is no longer growing. Thus, changes in k_i_ and K_i_ are likely to be only due to the regulation of enzymes and transporters. We chose to represent the putative variations of k_i_ and K_i_ during banana post-harvest ripening as a function of the number of days after ethylene treatment using the following mathematical expression:

$$k_{i,r}(t)=k_{i,r}*{\left(\frac{DAE}{DAE_{ref}}\right)}^{j_{i}}$$ (20)

$$K_{i,r}(t)=K_{i,r}*{\left(\frac{DAE}{DAE_{ref}}\right)}^{j_{i}}$$ (21)

where DAE is the day after ethylene treatment; DAE_ref_ is a reference day after ethylene treatment equal to 1; *k*_*i*,*r*_ (L day^-1^ or L^2^ day^-1^ mmol^-1^), *K*_*i*,*r*_ (L day^-1^), and *j*_*i*_ (dimensionless) are fixed parameters, with *k*_*i*,*r*_ and *K*_*i*,*r*_ positives, and *j*_*i*_ positive, null or negative. Depending on the values of the parameter *j*_*i*_, k_i,r_(t) and K_i,r_(t) can remain constant, increase, or decrease during ripening.

### Model inputs and initial conditions

The model used daily pulp fresh weight, and daily pulp structural dry weight as input variables. Initial values were the amount of citrate in the fruit pulp on the first date of the modeled period. Eq 17 was numerically solved using the ‘lsoda’ function of the package ‘dsolve’ of the R software with a one-day time step [36].

Daily pulp dry weight was estimated by fitting a growth expolinear function to pulp dry weight data [37].

$$DW=(\frac{C_{m}}{R_{m}})*\text{ln}(1+\text{exp}(R_{m}*(t-t_{b})))$$ (22)

where C_m_ is the maximum absolute growth rate of pulp dry weight (g day^-1^), R_m_ is the maximum relative growth rate of pulp dry weight (g g^-1^ day^-1^), t_b_ is the x axis intercept of the linear growth phase of pulp dry weight (day).

Daily pulp fresh weight was estimated using an empirical relationship with pulp dry weight (R² = 0.99 and *n* = 488):

FW=3.12*DW+3.47 (23)

Daily pulp structural dry weight was estimated using an empirical relationship with pulp dry weight (R² = 0.98 and *n* = 454):

$$SDW=0.69*DW^{0.73}$$ (24)

### Plant materials and experimental conditions

All experiments were conducted at the *Campus Agro-Environnemental Caraïbe* (CAEC, Martinique, French West Indies; latitude 14°37N, longitude 60°58W, altitude 16m) using three dessert banana cultivars (*Musa* spp.) diploids AA, that differ in their predominant organic acid at the eating stage: Indonesia 110 (IDN) which has a balanced ratio of citrate: malate, Pisang Jari Buaya (PJB) which is citrate dominant, and Pisang Lilin (PL) which is malate dominant (S1 Fig) [5]. In addition, these three cultivars present the advantage to be resistant to Black Leaf Streak Disease and Sigatoka Disease, two diseases known to affect banana fruit quality [38]. Experiments were conducted during the 2011 and 2012 growing seasons on continental alluvial soil. For the two growing seasons, irrigation was adjusted to the amount of rainfall to supply at least 5 mm of water per day, and non-systemic fungicide was applied to control foliar diseases. The mean daily temperature was 27 ± 1.2°C and 26± 0.9°C during the 2011 (March–November) and 2012 (February-August) growing season, respectively. In the 2011 experiment, 18 plants of each cultivar with high fruit load were used as the control treatment, and 18 other plants were highly pruned i.e. low fruit load. Fruit pruning was calculated so as to increase the leaf area: fruit ratio by approximately 2.5 for each cultivar (detailed in Etienne et al., 2014). In the 2012 experiment, two contrasted levels of potassium fertilization were applied: for each cultivar, one plot received 124 g of potassium per plant (high potassium fertilization) at 4-week intervals, while the other received no potassium at all (detailed in Etienne et al., 2014).

#### Fruit growth monitoring

In the two growing seasons, six bunches of each cultivar*treatment combination were selected. One fruit located in the internal row of the second proximal hand was collected for analyses every 15 days. Natural ripening on standing plants, i.e. when the first yellow finger appears, determined the end of sampling.

#### Monitoring of post-harvest ripening

Two harvest stages were studied in the 2011 experiment. The harvest stages were calculated for each cultivar to be 70% and 90% of the average flowering-to-yellowing time (FYT) of the bunch on the plant. For each harvest stage, six bunches of each cultivar*treatment combination were harvested. In the 2012 experiment, only one harvest stage was studied. The harvest stage was calculated for each cultivar to be 75% of the average FYT of the bunch on the plant. Six bunches of each cultivar*treatment combination were harvested. After the bunches were harvested, the second proximal banana hand per bunch was rinsed and dipped in fungicide (bitertanol, 200 mg L^−1^) for 1 min. The fruits were placed in a plastic bag with 20 μm respiration holes and stored in boxes for 6 days at 18°C. The fruits were then stored in a room at 18°C and underwent ethylene treatment (1 mL L^−1^ for 24 h) to trigger the ripening process. After 24 h, the room was ventilated. Bananas were maintained at 18°C for 13 days. A banana fruit was sampled before ethylene treatment, and at day 3, 6, 9 and 13 after ethylene treatment.

### Biochemical and respiration measurements

For each fruit sampled, the fresh and dried pulp were weighed. The dried pulp was then mixed to obtain dry powder and to allow biochemical measurements. Citrate concentration was assessed according to the method described in Etienne et al. [19] using an enzymatic method and a microplate reader. Pulp structural dry weight was calculated as the difference between pulp dry weight and the sum of the weights of the main non-structural compounds (soluble sugars, starch, acids). To this end, concentrations of malate were assessed according to the method described in Etienne et al. [19], and concentrations of starch and soluble sugars (glucose, fructose, sucrose) were assessed according to Gomez et al. [39], using an enzymatic method and a microplate reader. For measurements of respiration, each sampled fruit was placed in a closed plastic jar. The temperature of the room was set at 18°C. After 1 hour, CO_2_ concentration was measured with a gas analyzer (Vigaz, CANAL120).

### Model parameterization

Based on information found in the literature, the value of Cmal_cyt_ was set at 1 mM [40, 41], and the value of Cpyr_cyt_ was set at 0.5 mM [42, 43].

Parameters of the pulp dry weight growth model (C_m_, R_m_, t_b_) were estimated for each banana plant using a nonlinear least-squares regression method [44]. The model explained 99% of the pulp dry weight variance in the three cultivars and in the two years of the experiment, and the RRMSE was satisfactory with mean values of 0.1 (S2 Fig).

According to the literature, the temperature ratio of maintenance respiration Q_10_ was set at 2 [45]. The growth respiration coefficient of the pulp (q_g_) was derived from construction cost measurements on banana pulp. In the three cultivars, the total nitrogen, carbon and ash concentration of the banana pulp were measured at three different stages of fruit growth. The construction cost (CC; g glucose g^-1^) was calculated as a function of the carbon (C; g g DW^-1^), nitrogen (N; g g DW^-1^), and ash (A; g g DW^-1^) concentrations of banana pulp, and of the energetic costs of N assimilation and carbohydrate translocation [46, 47]:

$$CC=(5.39C+0.80A+5.64f_{Nh}N-1.191)(1+r_{T})$$ (25)

where f_Nh_ is the fraction of N used in growth that is assimilated heterotrophically, assumed to be equal to 1 for fruits [47], and r_T_ is the added cost of translocating photosynthetates from sources to sinks, assumed to be equal to 5.3% [46].

The coefficient q_g_ (mmol CO_2_ g^-1^) was calculated using the following formula [48]:

$$q_{g}=\frac{\left(\alpha CC-C\right)}{M_{C}}*1000$$ (26)

where αCC is the carbon construction cost (α = 0.4 is the concentration of carbon in glucose), and M_C_ is the molar mass of carbon equal to 12 g mol^-1^.

The calculated values of q_g_ were found to not significantly differ among cultivars and among developmental stages (data not shown). Thus, for q_g_ a value common to all cultivars and developmental stages was chosen as the mean value of the calculated values which was q_g_ = 13 mmol CO_2_ g^-1^ ±1. This value is in the range of values found for tomato (9.3 mmol CO_2_ g^-1^; [49]), peach (7.0 mmol CO_2_ g^-1^; [50]), and mango (3.0 mmol CO_2_ g^-1^; [48]).

The maintenance respiration coefficient (q_m_) of the pulp during banana growth and post-harvest ripening were calculated respectively from measurements of respiration in harvested green fruits and in fruits subjected to ethylene treatment in the 2012 experiment (*n* = 180). Since the fruits were harvested, we assumed that the measured respiration corresponded only to maintenance respiration. Therefore, by inverting Eq 16, q_m_ was calculated as follows:

$$q_{m}=\frac{Resp}{DWQ_{10}^{\frac{\theta -20}{10}}}$$ (27)

The value of q_m_ during fruit growth was estimated at q_m_ = 0.15 mmol CO_2_ g^-1^ day^-1^ ±0.02 for the three cultivars, a value close to those estimated for tomato (0.27 mmol CO_2_ g^-1^ day^-1^; [51]), peach (0.05 mmol CO_2_ g^-1^ day^-1^; [50]), and mango (0.09 mmol CO_2_ g^-1^ day^-1^; [48]).

The values of calculated q_m_ during postharvest ripening were plotted as a function of the number of days after ethylene treatment. In all three cultivars, q_m_ increased dramatically during the first two days after ethylene treatment, and then remained constant until the end of ripening (S3 A,S3 B and S3 C Fig). An appropriate expression to represent q_m_ was:

$$q_{m}=q_{m1}*(1-q_{m2}*{\text{exp}}^{-q_{m3}*DAE})$$ (28)

where DAE is the day after ethylene treatment, and q_m1_, q_m2_, and q_m3_ are fitted coefficients.

The values of q_m_ differed significantly among cultivars, so we decided to fit the model of Eq 28 to the three cultivars separately. Thus, parameters q_m1_, q_m2_, and q_m3_ were estimated for each cultivar by using a nonlinear least-squares regression method on the 2012 data [44] (Table 1). The model allowed satisfactory prediction of fruit respiration during postharvest ripening (R² = 0.72; RRMSE = 0.10) (S3 D Fig).

**Table 1:** Estimated parameter values and standard errors (in parentheses) of the q_m_ model during postharvest ripening in cultivars IDN, PJB, and PL.

	q_m1_ (mmol CO_2_ g^-1^ day^-1^)	q_m2_ (dimensionless)	q_m3_ (dimensionless)
IDN	0.61 (0.01)	0.71 (0.04)	1.08 (0.18)
PJB	0.48 (0.01)	0.68 (0.05)	0.65 (0.13)
PL	0.52 (0.01)	0.68 (0.04)	0.87 (0.13)

Parameters were estimated using the data from 2012 post-harvest ripening.

### Model calibration

Every parameters related to reaction rates k_i_(t) and membrane permeability K_i_(t) were estimated by fitting the predicted citrate concentrations to observed values of the 2011 dataset separately for each cultivar and developmental stage (60 data per cultivar during growth and 96 data per cultivar during post-harvest ripening) using the hydroPSO function of the R software [52]. The hydroPSO function uses the computational method of particle swarm optimization (PSO) that optimizes a problem by iteratively trying to improve a candidate solution with regard to a given measure of quality. Parameters were estimated by minimizing the following criterion:

$$\sum_{j}\sum_{i}{\left(\frac{x_{ij}-y_{ij}}{\sigma_{j}}\right)}^{2}$$ (29)

where x_ij_ is the predicted value, and y_ij_ is the observed value of the fruit of the j^th^ banana plant at date t_i_, and σ_j_ is the standard deviation of the observed values of the fruit of the j^th^ banana plant.

The model obtained, in which all the estimated parameters were different among cultivars, was named Model 1.

### Model selection

We used model selection to detect significant differences in parameter values among cultivars. Model 1 in which all the k_i,g_(t) and K_i,g_(t) were specific to cultivars were compared to reduced models in which some of the parameters were supposed to be the same for some of the cultivars, thus reducing the number of parameters to be estimated. For two models that do not significantly differ in fit quality, the one with fewer parameters is always preferred. The best model was named Model 2.

Models were selected using the Akaike information criterion (AIC), which allows the comparison of nested and non-nested models. In our case, the number of parameters exceeded *n*/40 (where *n* is sample size), so a second order derivative AICc, which contained a bias correction term for small sample size, was applied, as suggested by Johnson and Omland [53]. The model with smaller AICc was preferred.

$$AIC_{c}=n\;\text{ln}(\frac{RSS}{n})+2k(\frac{n}{n-k-1})$$ (30)

where n is the number of observations, RSS is the residual sum of squares, and k is the number of estimated parameters of the model.

### Sensitivity analysis of the model to pulp growth, respiration and temperature

A sensitivity analysis of the model was conducted in order to get insights into the effects of pulp growth, respiration and temperature on citrate accumulation during fruit growth and post-harvest maturation. We linked the pre- and post-harvest stages by taking into account the six days of fruit storage at 18°C between harvest and ethylene treatment, and assumed that, during that period, fruit respiration was equal to growth maintenance respiration. The sensitivity of the model was quantified by normalized sensitivity coefficients (SC), defined as the ratio between variation in the citrate concentration (ΔC) relative to its standard value (C), and variation in growth parameters, respiration parameters, and temperature (ΔP) relative to its standard value (P) [54].

$$\text{Normalized sensitivity coefficient}=\frac{\Delta C/C}{\Delta P/P}$$ (31)

The interpretation of SC is referred to as local sensitivity analysis since these coefficients provide information on the effect of small changes in the parameters (or inputs) on the response of the model. They do not provide information on the effect of simultaneous or large changes in parameters (or inputs). SCs were calculated by altering one parameter (or input) by ±0.1% while maintaining all the other parameters at default values. A positive and negative sign of SC correspond, respectively, to a response in the same or reverse direction as the variation in the parameter (or input). The larger the absolute value of SC, the more sensitive the model is to the parameter (or input). Since the SC behaved similarly between years for a given cultivar, only results in 2011 are presented. For the sensitivity analysis during postharvest ripening, the SCs behaved similarly between the two harvest stages in a given cultivar, so only results for 70% of FYT are presented.

### Goodness-of-fit and predictive quality of the model

The goodness-of-fit of the model was evaluated using two commonly used criteria, the root mean squared error (RMSE) and the relative root mean squared error (RRMSE), to compare the mean difference between simulated and observed results [55]. The smaller the value of RMSE and RRMSE, the better the fit.

$$RMSE=\sqrt{\frac{\sum {\left(Y_{i}-X_{i}\right)}^{2}}{n}}$$ (32)

where Y_i_ is the predicted value of the fruit i, and X_i_ is the measured value of the fruit i. n is the number of data.

$$RRMSE=\frac{RMSE}{\bar{X}}$$ (33)

where $\bar{\text{x}}$ is the mean of all observed values.

The predictive quality of the model, which ascertains the validity of the model in different scenarios, was quantified by the RMSE and RRMSE calculated on the 2012 data set.

## Results

### Overview of cultivar and treatment effects

**Fig 3:**
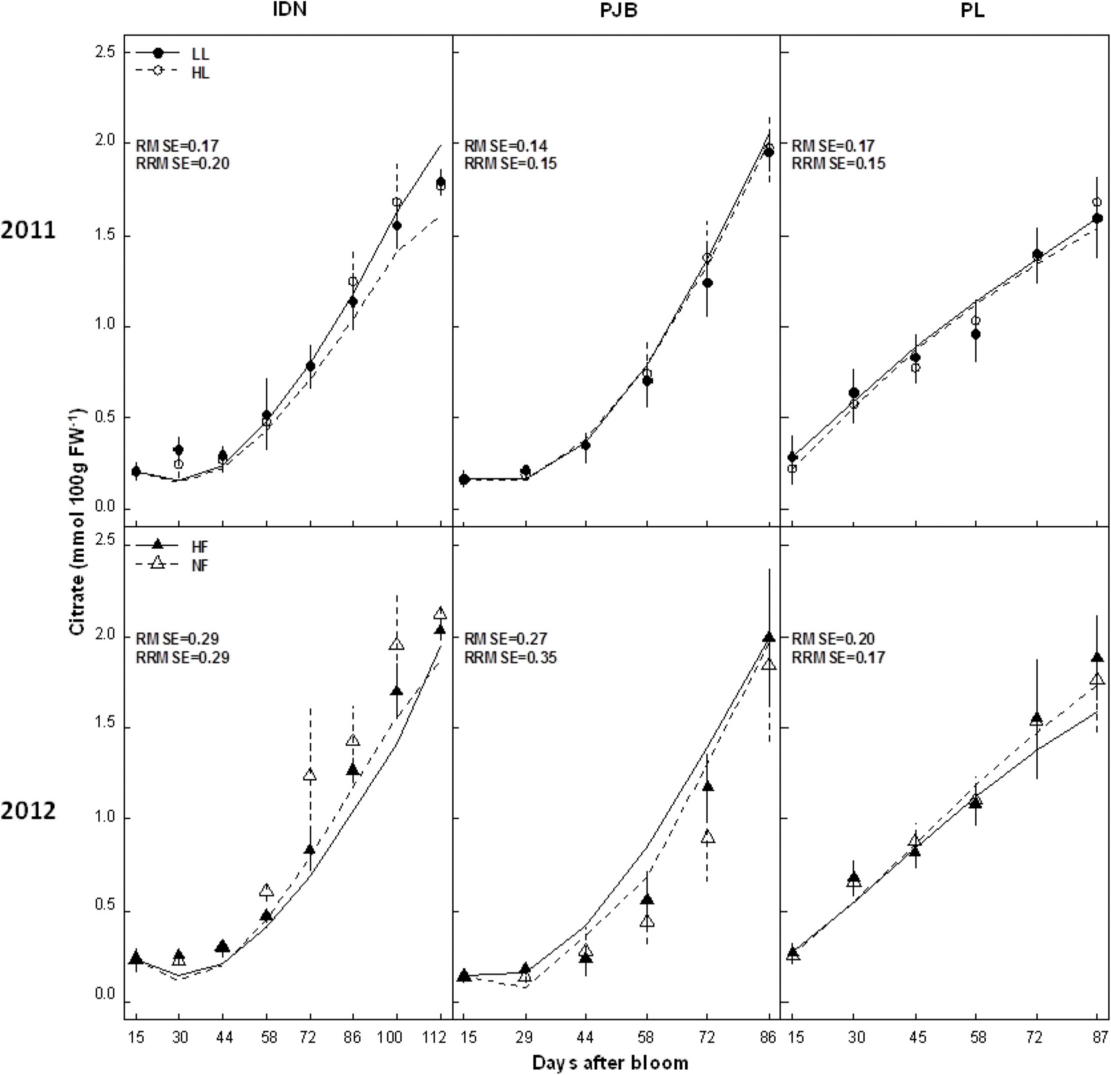
Measured (dots) and simulated (lines) citrate concentrations in the pulp of cultivars IDN, PJB, and PL during fruit growth. The cultivars were grown under two contrasted fruit loads in 2011 (LL: low fruit load; HL: high fruit load), and two contrasted levels of potassium fertilization in 2012 (NF: no potassium fertilization; HF: high level of potassium fertilization). Data are means ± s.d (*n* = 6). The RMSE (mmol 100g FW^*-1*^) and RRMSE are indicated in each graph.

**Fig 4:**
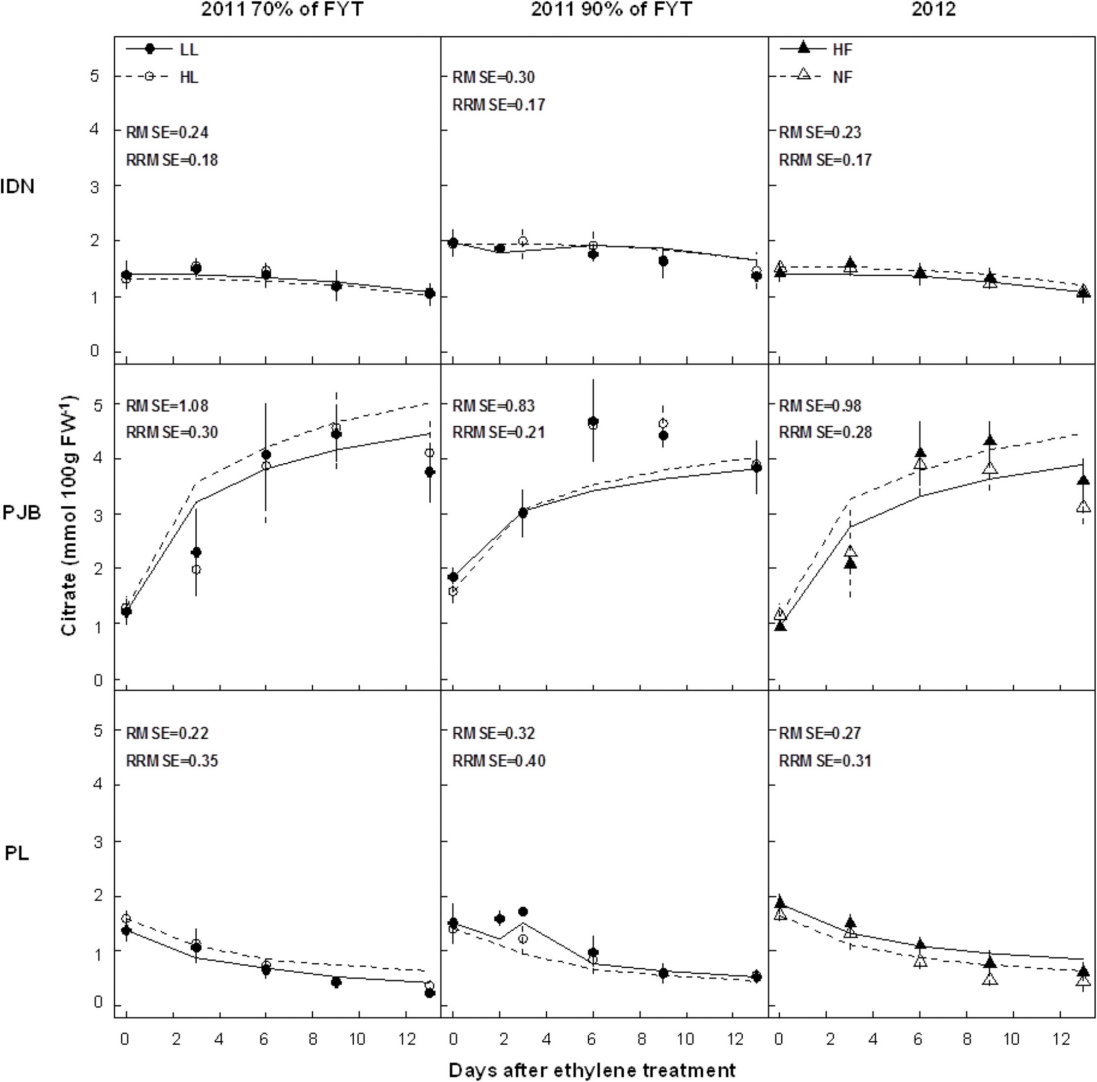
Measured (dots) and simulated (lines) citrate concentrations in the pulp of cultivars IDN, PJB, and PL during fruit post-harvest ripening. The cultivars were grown under two contrasted fruit loads in 2011 (LL: low fruit load; HL: high fruit load), and two contrasted levels of potassium fertilization in 2012 (NF: no potassium fertilization; HF: high level of potassium fertilization). In 2011, fruits were harvested at two different stages: early stage (70% of FYT) and late stage (90% of FYT). Data are means ± s.d (*n* = 6). The RMSE (mmol 100g FW^*-1*^) and RRMSE are indicated in each graph.

The effects of cultivar and treatments on citrate concentration in banana pulp during the pre- and post-harvest stages are detailed in a previous paper [20], so only the main conclusions are presented here. During banana growth, citrate concentration increased and was significantly affected by cultivar both in 2011 and 2012. The PL cultivar had significantly higher citrate concentration in the pulp than the IDN and PJB cultivars (Fig 3). During banana post-harvest ripening, both the ripening stage and the cultivar had a significant effect on the concentrations of citrate in 2011 and 2012. The pattern of citrate concentration in the pulp were the same in the IDN and PL cultivars with an overall decrease during ripening, whereas there was an overall increase in the PJB cultivar (Fig 4). At the end of ripening, PJB had the highest citrate concentration and PL the lowest. Fruits harvested later (at 90% of flowering-to-yellowing time (FYT)) had significantly higher concentration of citrate throughout ripening in the three cultivars. Low fruit load significantly increased the pulp fresh weight in all three cultivars but had no effect on the concentration of citrate during either growth or post-harvest ripening. Potassium fertilization significantly increased the pulp fresh weight in cultivars PJB and PL, whereas the opposite was the case for fruits from the IDN cultivars. However, potassium fertilization had no effect on the concentration of citrate during either growth or post-harvest ripening in all three cultivars.

### Model calibration and selection

Model 1 was fitted to observed data by estimating 48 parameters (16 per cultivar), and predictions were in good agreement with observed 2011 data for the three cultivars (mean RRMSE = 0.23). Comparison of model 1 with several reduced models was made based on the AICc criterion (data not shown). We selected the reduced model 2. In model 2, during the pre-harvest stage, *m*_*1*_, *m*_*3*_ and *m*_*4*_ were fixed at 0 for the three cultivars; *k*_*1*,*g*_, *k*_*3*,*g*_, *K*_*4*,*g*_ were the same for the three cultivars; and *K*_*5*,*g*_ and *m*_*5*_ were specific to cultivars, thus reducing the number of estimated parameters to nine (Table 2). During the post-harvest stage, *j*_*1*_ were fixed at 0 for PJB, *j*_*3*_ and *j*_*4*_ were fixed at 0 for the three cultivars, *j*_*5*_ were fixed at 0 for IDN and PL; and *K*_*4*,*r*_ and *K*_*5*,*r*_ were the same for cultivars IDN and PL, reducing the number of estimated parameters to 13 (Table 2). Values of the 22 estimated parameters of model 2 are summarized in Table 3.

**Table 2:** Results of model selection between full and reduced models using AICc criteria.

Model	AICc	Stage	Estimated parameters	Fixed parameters
1	-665	Pre-harvest	k_1,g_^idn^,m_1_^idn^,k_3,g_^idn^,m_3_^idn^,K_4,g_^idn^,m_4_^idn^,K_5,g_^idn^,m_5_^idn^,k_1,g_^pjb^,m_1_^pjb^,k_3,g_^pjb^,m_3_^pjb^,K_4,g_^pjb^,m_4_^pjb^,K_5,g_^pjb^,m_5_^pjb^,k_1,g_^pl^,m_1_^pl^,k_3,g_^pl^,m_3_^pl^,K_4,g_^pl^,m_4_^pl^,K_5,g_^pl^,m_5_^pl^	
		Post-harvest	k_1,r_^idn^,j_1_^idn^,k_3,r_^idn^,j_3_^idn^,K_4,r_^idn^,j_4_^idn^,K_5,r_^idn^,j_5_^idn^, k_1,r_^pjb^,j_1_^pjb^,k_3,r_^pjb^,j_3_^pjb^,K_4,r_^pjb^,j_4_^pjb^,K_5,r_^pjb^,j_5_^pjb^, k_1,r_^pl^,j_1_^pl^,k_3,r_^pl^,j_3_^pl^,K_4,r_^pl^,j_4_^pl^,K_5,r_^pl^,j_5_^pl^	
2	-729	Pre-harvest	k_1,g_^idn^ = k_1,g_^pjb^ = k_1,g_^pl^,k_3,g_^idn^ = k_3,g_^pjb^ = k_3,g_^pl^, K_4,g_^idn^ = K_4,g_^pjb^ = K_4,g_^pl^, K_5,g_^idn^,m_5_^idn^,K_5,g_^pjb^,m_5_^pjb^,K_5,g_^pl^,m_5_^pl^	m_1_^idn^ = m_1_^pjb^ = m_1_^pl^ = 0, m_3_^idn^ = m_3_^pjb^ = m_3_^pl^ = 0, m_4_^idn^ = m_4_^pjb^ = m_4_^pl^ = 0
		Post-harvest	k_1,r_^idn^,j_1_^idn^,k_3,r_^idn^,k_1,r_^pl^,j_1_^pl^,k_3,r_^pl^, k_1,r_^pjb^,k_3,r_^pjb^,K_4,r_^pjb^,K_5,r_^pjb^,j_5_^pjb^, K_4,r_^idn^ = K_4,r_^pl^,K_5,r_^idn^ = K_5,r_^pl^	j_1_^pjb^ = 0,j_5_^idn^ = j_5_^pl^ = 0, j_4_^idn^ = j_4_^pjb^ = j_4_^pl^ = 0, j_3_^idn^ = j_3_^pjb^ = j_3_^pl^ = 0

The superscript names following the parameters refer to banana cultivars IDN, PJB and PL. The best model is Model 2.

**Table 3:** Selected rate constants and membrane permeability for the cultivars IDN, PJB, and PL according to the best model (Model 2).

Rate constants (k_i_) and membrane permeability (K_i_)	Unit	Equation		
		PL	IDN	PJB
Pre-harvest				
k_1,g_(t)	L².day^-1^.mmol^-1^	9000		
k_3,g_(t)	L.day^-1^	2		
K_4,g_(t)	L.day^-1^	4000		
K_5,g_(t)	L.day^-1^	0.0048*SDW(t)^0.98^	0.0012*SDW(t)^1.99^	0.0017*SDW(t)^1.95^
Post-harvest				
k_1,r_(t)	L².day^-1^.mmol^-1^	5634*t^1.36^	4904*t^-1.99^	9887
k_3,r_(t)	L.day^-1^	531	0.17	0.08
K_4,r_(t)	L.day^-1^	3965		6889
K_5,r_(t)	L.day^-1^	1.0*10^–4^		1.03*t^-1.26^

### Evaluation of the model

Citrate concentrations simulated by model 2 were in good agreement with 2011 values observed in the three cultivars (RMSEs and RRMSEs ranged between 0.14 and 1.08 mmol 100g FW^-1^, and between 0.15 and 0.40 respectively) (Figs 3 and 4). Model validation on data from 2012 was also satisfactory, as revealed by RMSEs and RRMSEs of predictions, with values ranging between 0.20 and 0.98 mmol 100g FW^-1^, and between 0.17 and 0.35 respectively. Statistical analysis revealed that the model correctly simulated the strong effect of cultivar and fruit age (S2 and S3 Tables). The model predicted little effect of fruit load and potassium fertilization, consistent with observed data. The model predicted a small effect of harvest stage, consistent with observed data, but was not able to simulate the minor differences correctly (data not shown).

### Predictions of metabolic fluxes

**Fig 5:**
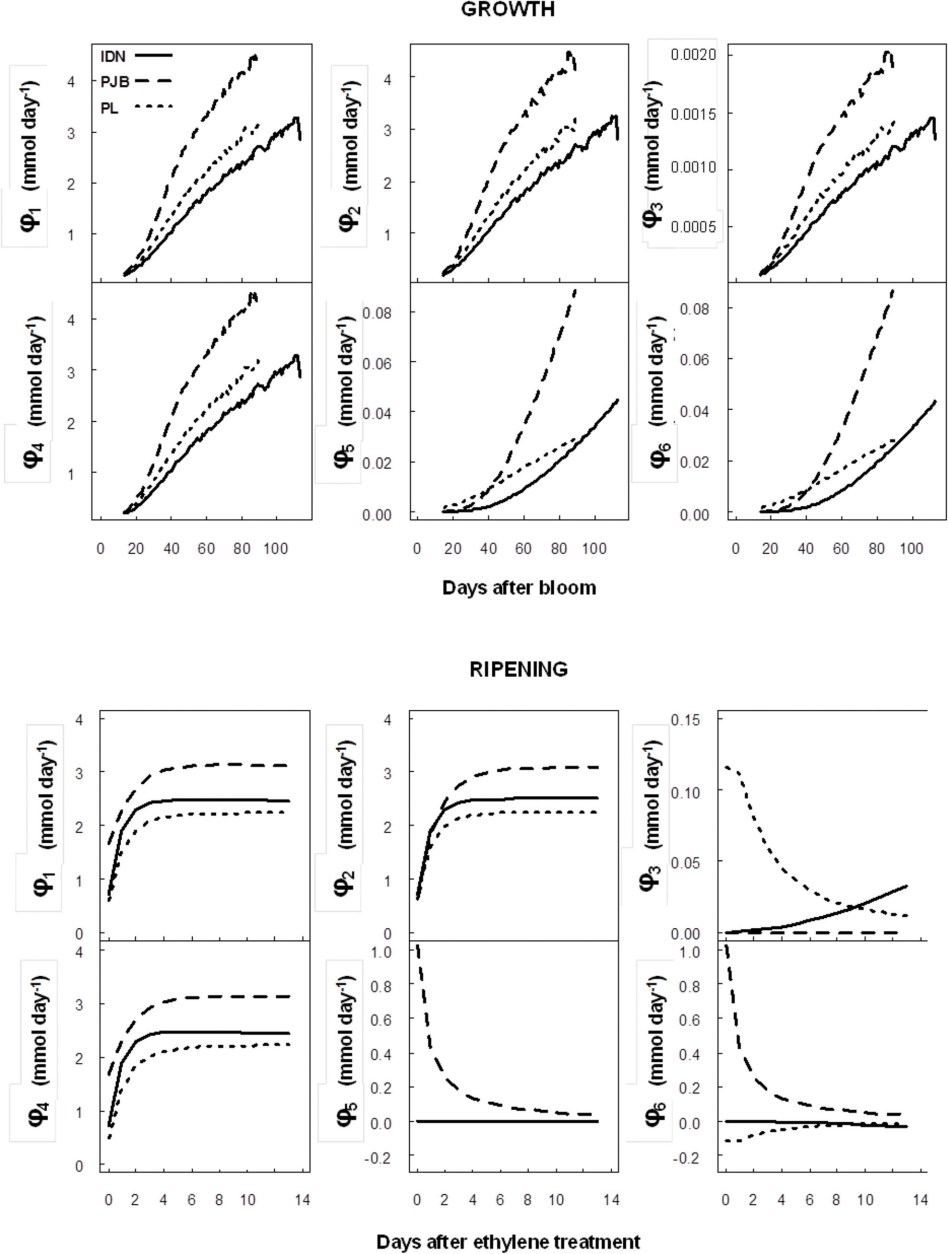
Metabolic fluxes of the TCA cycle predicted by the citrate model during fruit growth and post-harvest ripening for the cultivars IDN, PJB, and PL.

The metabolic fluxes of the TCA cycle predicted by the model are presented in Fig 5 for the three cultivars. During banana growth, all the metabolic fluxes underwent a continuous increase in the three cultivars. φ_1_, φ_2_, φ_3_, and φ_4_ followed the same pattern, but φ_3_ was a lot lower. φ_5_ and φ_6_ followed another pattern. All the metabolic fluxes were highest in cultivar PJB, then in cultivar PL and lastly in cultivar IDN. During post-harvest ripening, φ_1_, φ_2_, and φ_4_ dramatically increased in the first two days after ethylene treatment and then remained constant in all three cultivars. φ_1_, φ_2_, and φ_4_ were highest in cultivar PJB, then in cultivar IDN and lastly in cultivar PL. There were great differences in the pattern of φ_3_, φ_5_, and φ_6_ among cultivars during ripening. φ_3_ was close to zero for PJB, was positive and increased for IDN, and was positive and decreased for PL. φ_5_ and φ_6_ were close to zero for IDN and PL, and were high for PJB at the beginning of ripening but then decreased to 0.

### Sensitivity analysis to growth, respiration and temperature

**Fig 6:**
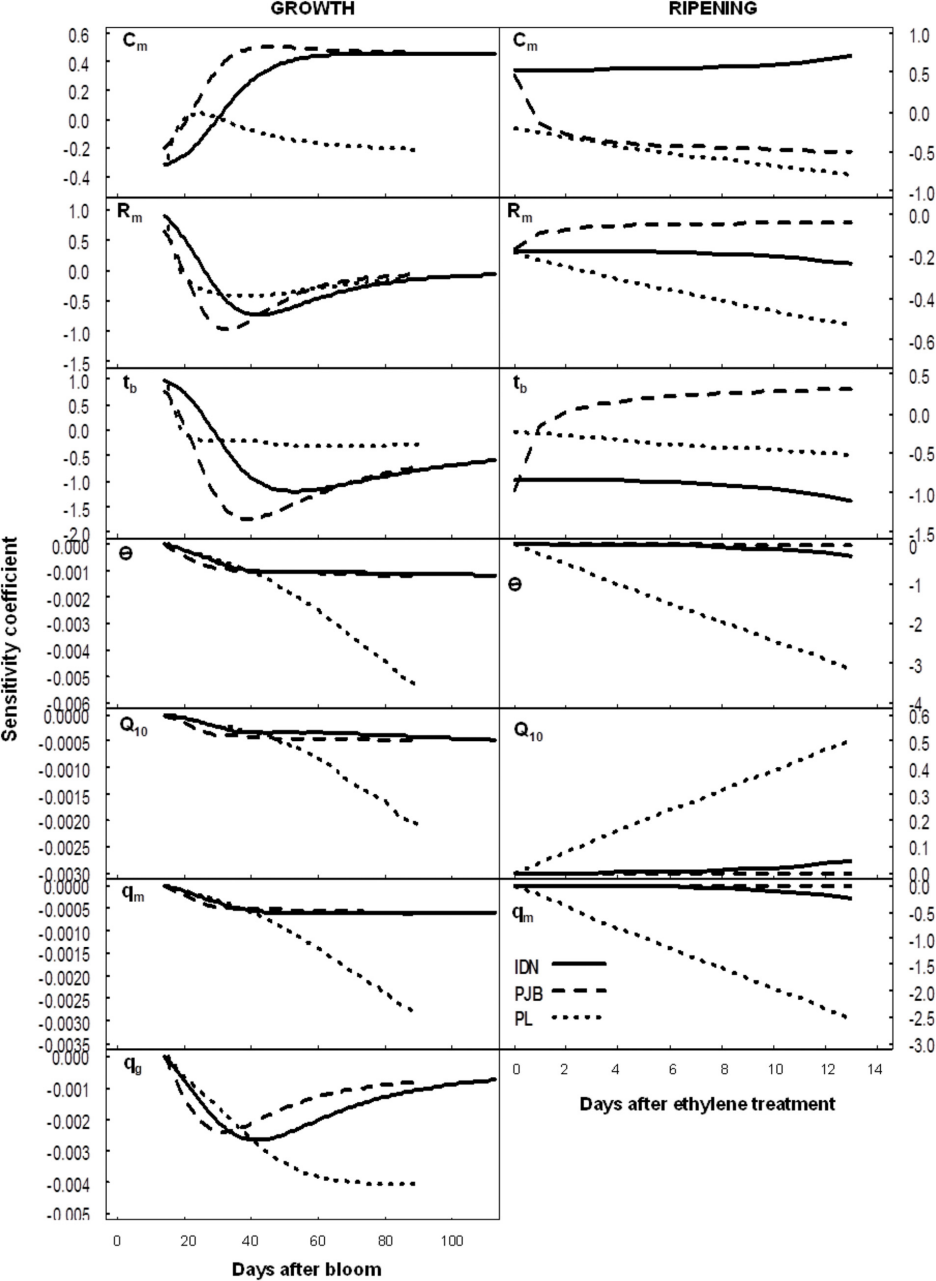
Normalized sensitivity coefficients of citrate concentration to the growth parameters, temperature, and respiration parameters during growth and post-harvest ripening in the cultivars IDN, PJB, and PL. Temperature refers to air temperature during fruit growth, and to storage temperature during fruit ripening.

During banana growth, pulp growth parameters influenced citrate concentration in a cultivar dependent manner (Fig 6). The sensitivity of the model to C_m_ was higher in cultivars IDN and PJB, than in cultivar PL. The SC of C_m_ increased in the first part of growth, and remained almost constant thereafter. The model was very sensitive to R_m_ and t_b_ at the beginning of growth, and less at the end. During postharvest ripening, C_m_ had a positive effect on citrate concentration in the cultivar IDN, and a negative effect in cultivars PJB and PL. R_m_ had a negative effect on citrate concentration in all three cultivars. t_b_ had a negative effect on citrate concentration in cultivar IDN, and a positive effect in cultivars PJB and PL.

During banana growth, respiration parameters and temperature had no effect on citrate concentration in any of the three cultivars (Fig 6). During post-harvest ripening, q_m_ and storage temperature greatly influenced citrate accumulation in a negative way in cultivar PL but had no effect in cultivars IDN and PJB. Q_10_ had a positive effect on citrate accumulation in cultivar PL, and no effect in cultivars IDN and PJB. The reason why Q_10_ had a positive effect was that storage temperature was lower than 20°C (see Eq 16).

## Discussion

### Metabolism more than dilution by growth was responsible for the genotypic variability in citrate concentration

The concentration of citrate in fruit pulp is the result of several processes. First, the metabolic processes of citrate control the amount of citrate in the pulp (for review see [9]). Second, the accumulation of water and dry matter in the fruit influences the concentration of citrate in the pulp due to dilution caused by change in fruit volume [56]. Pulp fresh weight increased during the pre-harvest stage for the three cultivars [20] but not sufficiently to induce a decrease in the concentration of citrate in the pulp. Thus, during the pre-harvest stage, citrate concentration was mainly linked to its metabolism. This result is in accordance with Wu et al. [18] who showed that peach citrate concentration was mainly linked to its metabolism during the green stage. As a consequence, the differences in the concentration of citrate in banana pulp among cultivars were mainly the result of differences in metabolism rather than in dilution [20]. During post-harvest ripening, since pulp fresh weight did not change anymore, the main component affecting citrate concentration and consequently responsible for genotypic variability was metabolism.

### Potential role for NAD-malic enzyme and mitochondrial malate carriers in the genotypic variability of citrate metabolism during the pre- and post-harvest stages

**Fig 7:**
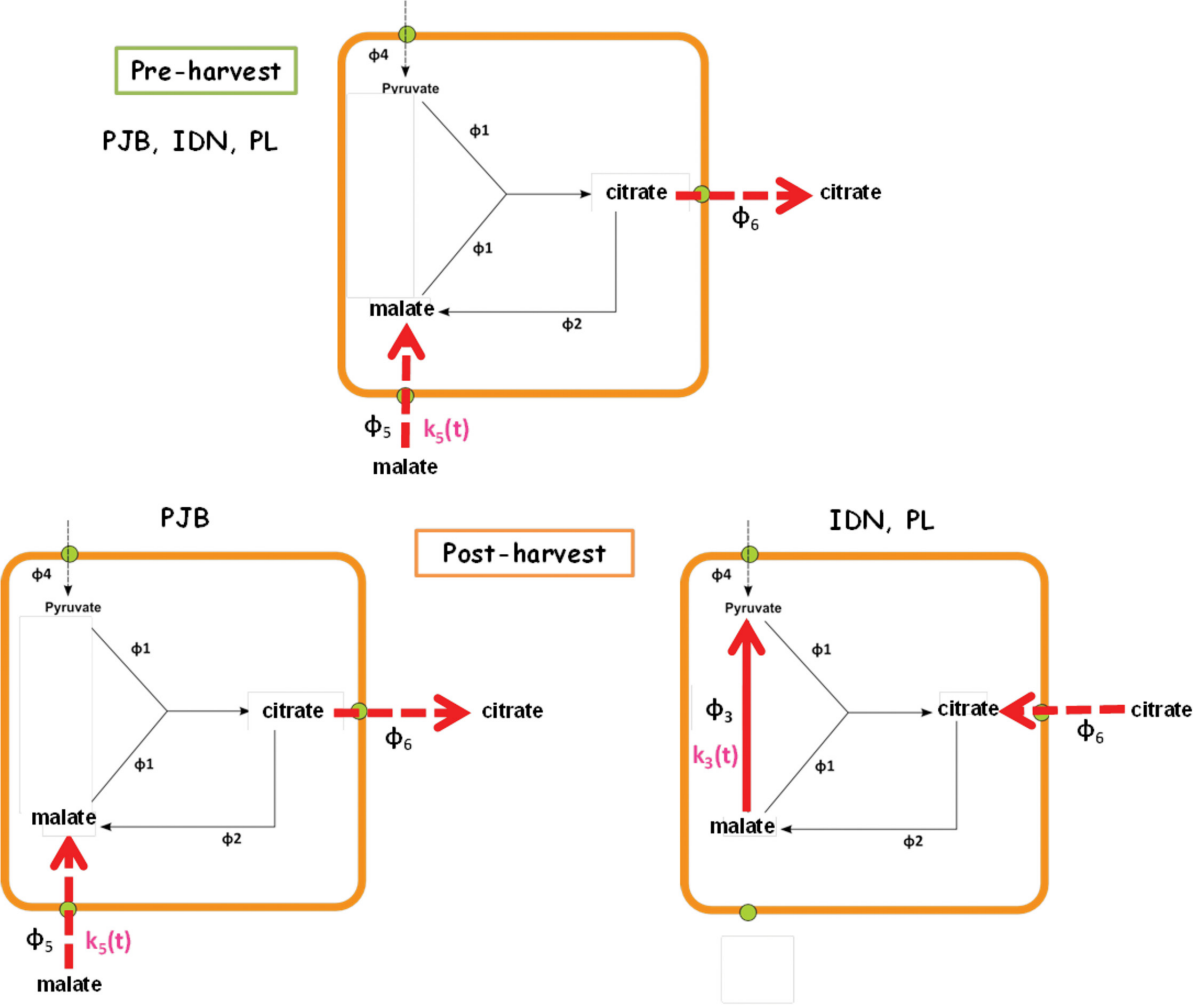
Schematic diagram of the differences in organic acid metabolism predicted by the model among the three cultivars during the pre and post-harvest stages. Red arrows indicate major fluxes involved in citrate accumulation. The genotypic parameters identified by the model are represented in pink.

The model gives a possible explanation for differences in citrate metabolism between the pre- and post-harvest stages, and among cultivars (Fig 7 *)*. During fruit growth, the model predicted a common metabolic scheme for the three cultivars with the flux of malate conversion into pyruvate (φ_3_) close to zero, and consequently the flux of net citrate production (φ_6_) was equal to the flux of malate imported into mitochondria (φ_5_). Thus, during the pre-harvest stage, the import of malate into the mitochondria drove citrate production. The model suggested that differences in citrate accumulation among cultivars during fruit growth were due to differences in φ_5_, and in particular in K_5,g_(t), which reflects the activity of malate mitochondrial transporters. In the three cultivars, K_5,g_(t) increased during banana growth but increased more in cultivar PJB than in the two other cultivars. It is known that the expression of mitochondrial malate transporters varies during fruit development [31]. The absence of φ_3_ suggests that the pyruvate imported into the mitochondria through φ_4_ could completely satisfy the fruit energy demand, with no need to produce any pyruvate via the mitochondrial malic enzyme reaction.

During post-harvest ripening, the model predicted the same metabolic scheme as during growth in the cultivar PJB, i.e. the import of malate into the mitochondria drove citrate production. In cultivar PJB, K_5,r_(t) was important at the beginning of ripening and then decreased dramatically, explaining why the concentration of citrate increased significantly in the first days after ethylene treatment and then remained almost constant. In cultivars IDN and PL, φ_3_ was positive and φ_5_ was equal to zero during post-harvest ripening. Consequently, φ_6_ was equal to-φ_3_ and was therefore negative (in particular for PL; φ_6_ was close to zero for IDN), explaining the decrease in citrate concentration. Model selection indicated that k_3,r_(t), which reflects mitochondrial NAD-malic enzyme activity, can be assumed to remain constant during post-harvest ripening in the three cultivars, suggesting that NAD-malic enzyme activity is likely to vary little during this stage. Borsani et al. [33] observed no significant changes in NAD-malic enzyme activity during post-harvest ripening in peach. Differences in citrate concentrations between cultivars IDN and PL were due to differences in φ_3_, and in particular in k_3,r_(t). There is no information in the literature concerning the possible involvement of mitochondrial NAD-malic enzyme in the difference in acidity among fruit cultivars, but the model suggests this could be an interesting avenue to explore. In the end, it appears that screening for a genotype with low mitochondrial malate transport activity or high mitochondrial NAD-malic enzyme activity could be the most promising way to achieve a low citrate concentration in ripe fruit.

### Pulp growth affected citrate metabolism in a cultivar-dependent manner

**Fig 8:**
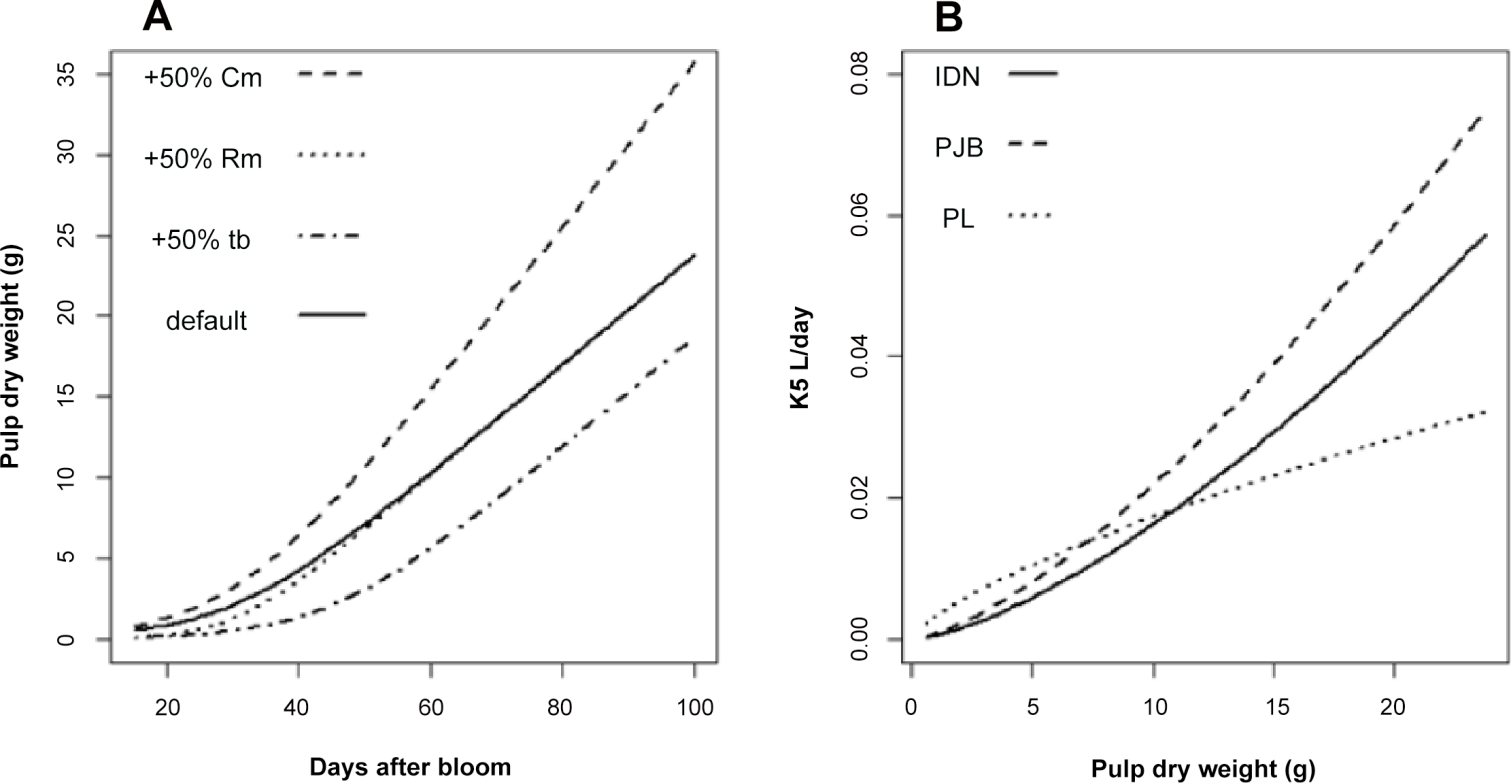
Changes in pulp dry weight in response to (A) growth parameters and (B) K_5,g_(t) for the three cultivars. Growth parameters were increased one at a time by 50% of their default value. K_**5,g**_(t) were calculated for each cultivar using the equations presented in Table 3.

The effect of fruit load on citrate concentration predicted by our model was the result of modifications in banana pulp growth (S4 Table). Increasing pulp growth parameters affected pulp dry weight, and consequently pulp fresh weight, in different ways (Fig 8A) with consequences on citrate metabolism. During fruit growth, φ_6_ was equal to φ_5_ for the three cultivars, which means that φ_6_ depended on K_5,g_(t) and Mal_mt_ (see Eq 9). Increasing pulp dry weight increased K_5,g_(t) (Fig 8B). Mal_mt_ was positively related to dry weight through respiration. Nevertheless since Mal_mt_ was negligible compared to Mal_cyt_ (data not shown), respiration had no impact on φ_6_. Therefore, increasing pulp dry weight increased the weight of citrate in the pulp due to the increase in K_5,g_(t). In the end, the effect of pulp growth parameters on citrate concentration in the pulp depended on whether the effect on citrate production was greater than the effect on fresh weight (or not), explaining why the sensitivity coefficients of citrate concentration in the pulp varied so much during fruit growth. Wu et al. [18] used the citrate model of Lobit et al. [17] to study the effect of peach mesocarp growth on citrate concentration. They showed that an increase of pulp dry weight increased both fresh weight and citrate production, which increased citrate concentration at the beginning of fruit development and decreased it near maturity depended on whether the effect on citrate production was greater than the effect on fresh weight (or not). The fact that respiration had no impact on banana citrate concentration during the pre-harvest stage explains why the SCs of respiration parameters were close to zero which was similar to the results on peach maintenance respiration parameters obtained by Wu et al. [18]. However our results on banana growth respiration coefficient contrasted with the results on peach which showed that increasing growth respiration coefficient increased citrate concentration in particular at the beginning of fruit growth [18].

During post-harvest ripening, bigger fruits had higher respiration which had cultivar-dependent consequences on post-harvest citrate metabolism. For PL, φ_6_ was equal to-φ_3_ and was therefore negative. Since φ_3_ was positively related to Mal_mt_, itself positively related to respiration, fruits with higher respiration had higher rate of citrate degradation. This explains why increasing q_m_, as well as decreasing Q_10_, decreased citrate concentration. For IDN, φ_6_ was close to zero and was therefore not affected by respiration. For PJB, φ_6_ was equal to φ_5_. Therefore, increasing respiration had no effect on citrate production because K_5,r_(t) only depended on time during post-harvest ripening (see Eq 21). The effect of pulp growth parameters on the concentration of citrate in the pulp during post-harvest ripening depended on their effects on citrate concentration at harvest (i.e. during the pre-harvest stage), on citrate metabolism after ethylene treatment and on fresh weight. All these combined effects explain why the SCs of citrate concentration to pulp growth parameters changed during post-harvest ripening. The minor effect of potassium fertilization on citrate concentration predicted by the model during post-harvest ripening was the result of small modifications in pulp growth. Fruits grown under contrasted potassium fertilization had different growth parameters (S5 Table) that affected citrate concentration during post-harvest ripening in a cultivar dependent manner.

### The model predicted a cultivar-dependent response of citrate concentration to storage temperature

During fruit growth, the model predicted no effect of temperature on the concentration of citrate in the pulp for the three cultivars, whereas it showed a cultivar-dependent response to storage temperature during post-harvest ripening. A negative effect of storage temperature was predicted on the concentration of citrate for cultivar PL but none for cultivars IDN and PJB. This is an interesting outcome of the model since storage temperature is a variable easy to control, however these results need to be checked experimentally. The negative effect of temperature on citrate concentration predicted for cultivar PL is in accordance with the fact that, as frequently observed in field experiments, high temperatures reduce fruit acidity (for review see [9]). Moreover, Bugaud et al. [57] observed that banana harvested during the cold season had the highest concentration of citrate. The cultivar dependency of citrate concentration to temperature has also been simulated by Wu et al. [18] on peach using the model of Lobit et al. [17]. This model predicted that raising mean temperature during ripening increased citrate concentration for cv. Fidelia whereas it had almost no effect for cv. Suncrest. The main reason was that for cv. Fidelia, a parameter k_3_ related to temperature was additionally included in the model, compared with the model for cv. Suncrest. In our model, temperature affected citrate concentration only through modification of respiration. We did not consider any effect of temperature on enzyme and transporter activities but it is known that temperature controls the reaction rates of glycolysis and of the TCA cycle [35] by modifying enzyme activities [58], and also the kinetic properties of the mitochondrial transport systems involved [59]. Therefore, a possible improvement of the citrate model could be to take into account the temperature dependency of enzyme and transporter activity.

### Model validity

We assumed that citrate accumulation is driven by the TCA cycle located in the mitochondria. This assumption is in fact a simplification of the complex carbon metabolism that takes place in fleshy fruit (for review see [9]). The model was based on a simplified representation of the TCA cycle and several processes were not considered, like the regulation of TCA cycle by coenzymes among others. The validity of flux predictions could be tested by comparing them to experimental measurements of metabolic fluxes. This kind of approach is commonly used to model metabolic flux and helps constrain the flux solution space [60]. Moreover, Sweetlove et al. [60] suggested that metabolic input and output are the key drivers of flux distribution, more than enzyme regulation. Consequently, the precise determination of the input metabolites considered in our model (cytosolic malate and pyruvate concentrations) should help improve metabolic predictions. In the present model, cytosolic malate and pyruvate concentrations were assumed to be constant during banana development, whereas in reality they certainly fluctuate since they play a role in the regulation of cytosolic pH [61]. Further work will be required to validate the main conclusions of the model with experimental results (i.e. measurements of enzyme activities or mRNA levels), as e.g. the possible involvement of mitochondrial NAD-malic enzyme in the difference in acidity among fruit cultivars.

## Conclusion

The model of TCA cycle functioning proposed in this study satisfactorily simulated the dynamics of citrate concentration during the pre- and post-harvest stages of fruit development in a range of cultivars. This model turned out to be an interesting tool to dissect the effects of genotypic and environmental factors on citrate concentration. In particular, the model suggested major differences in TCA cycle functioning among cultivars during post-harvest ripening of banana, and pointed to the potential role of NAD-malic enzyme and mitochondrial malate carriers in the genotypic variability of citrate concentration. The next step will be to validate these assumptions experimentally. Given that the TCA cycle is a universal feature of fruit metabolism, the present model can be used as a conceptual basis for modeling citrate accumulation in other fruit species. In the future, linking such models with a model for malate [22], and a model relating titratable acidity and pulp composition [19], would be a powerful tool for fruit quality improvement.

## Supporting Information

S1 Dataset An excel file containing the data used for model calibration and validation. (XLSX)

S1 Fig Pictures of the fruits of three cultivars of dessert bananas used in the 2011 and 2012 field experiments and graphics showing the repartition of the different organic acids present in the pulp of ripe banana fruit. (TIF)

S2 Fig Observed pulp dry weight vs. pulp dry weight predicted by the growth expolinear model for cultivars IDN, PJB, and PL in 2011 and 2012. (TIF)

S3 Fig Variations in the maintenance respiration coefficient q_m_ during post-harvest ripening of banana in cultivars IDN, PJB, and PL, and predicted vs. measured fruit respiration. (TIF)

S1 Table Description and units of parameters, constants and variables. (PDF)

S2 Table LMM analysis of predicted and measured citrate concentration during fruit growth. (PDF)

S3 Table LMM analysis of predicted and measured citrate concentration during postharvest fruit ripening. (PDF)

S4 Table Estimated parameter values and standard errors of the expolinear growth model of pulp dry weight for the three cultivars and two contrasted fruit loads in 2011. (PDF)

S5 Table Estimated parameter values and standard errors of the expolinear growth model of pulp dry weight in the three cultivars and the two contrasted levels of potassium fertilization in 2012. (PDF)
