# Supplementary figures and images for: A Process-Based Model of TCA Cycle Functioning to Analyze Citrate Accumulation in Pre- and Post-Harvest Fruits
Source: PLoS One. 2015 Jun 4;10(6):e0126777. doi: 10.1371/journal.pone.0126777 (PMC4456289; doi:10.1371/journal.pone.0126777)

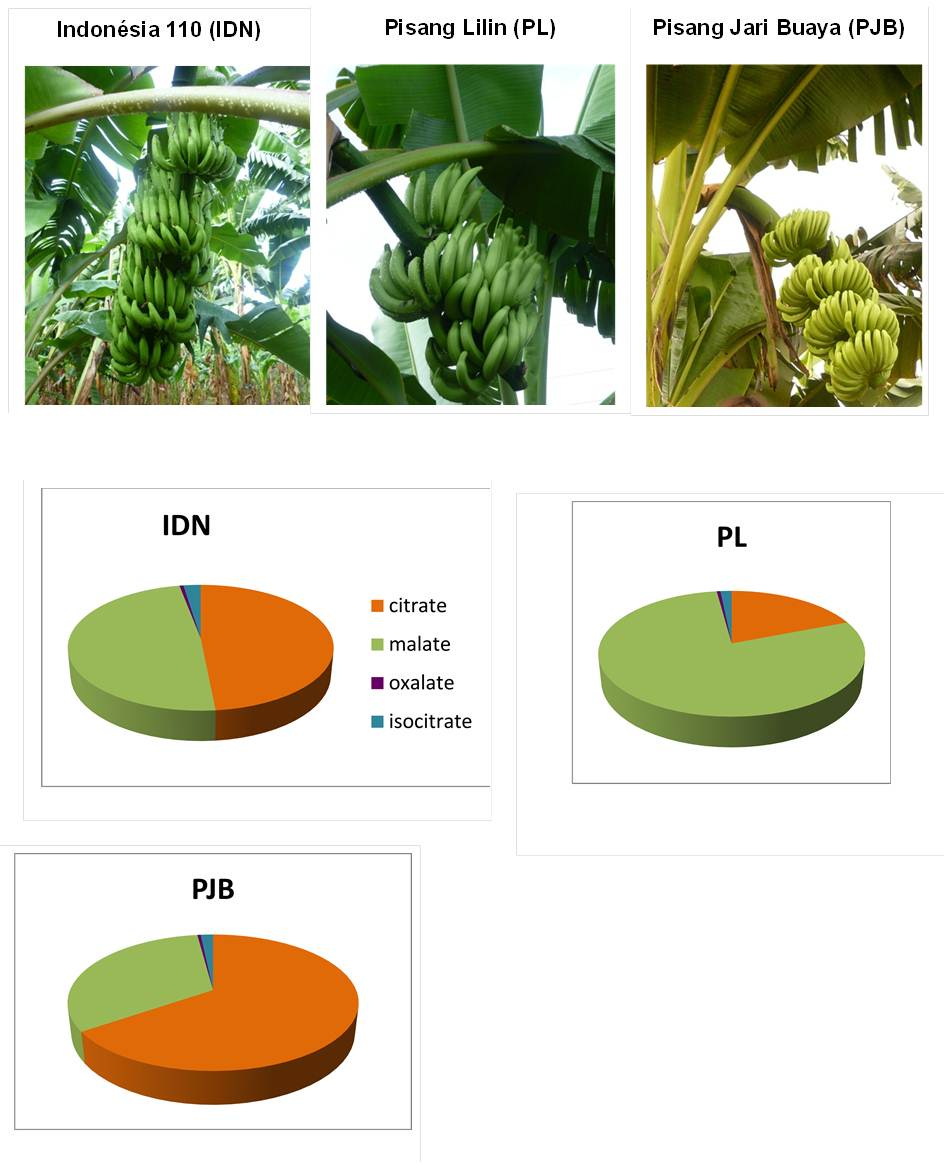

Supplement: S1 Fig — (TIF) [file pone.0126777.s002.tif]

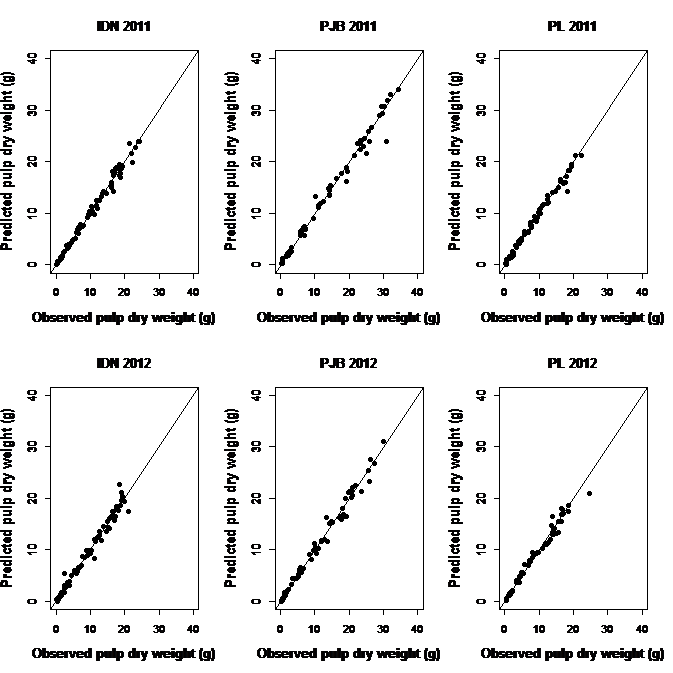

Supplement: S2 Fig — (TIF) [file pone.0126777.s003.tif]

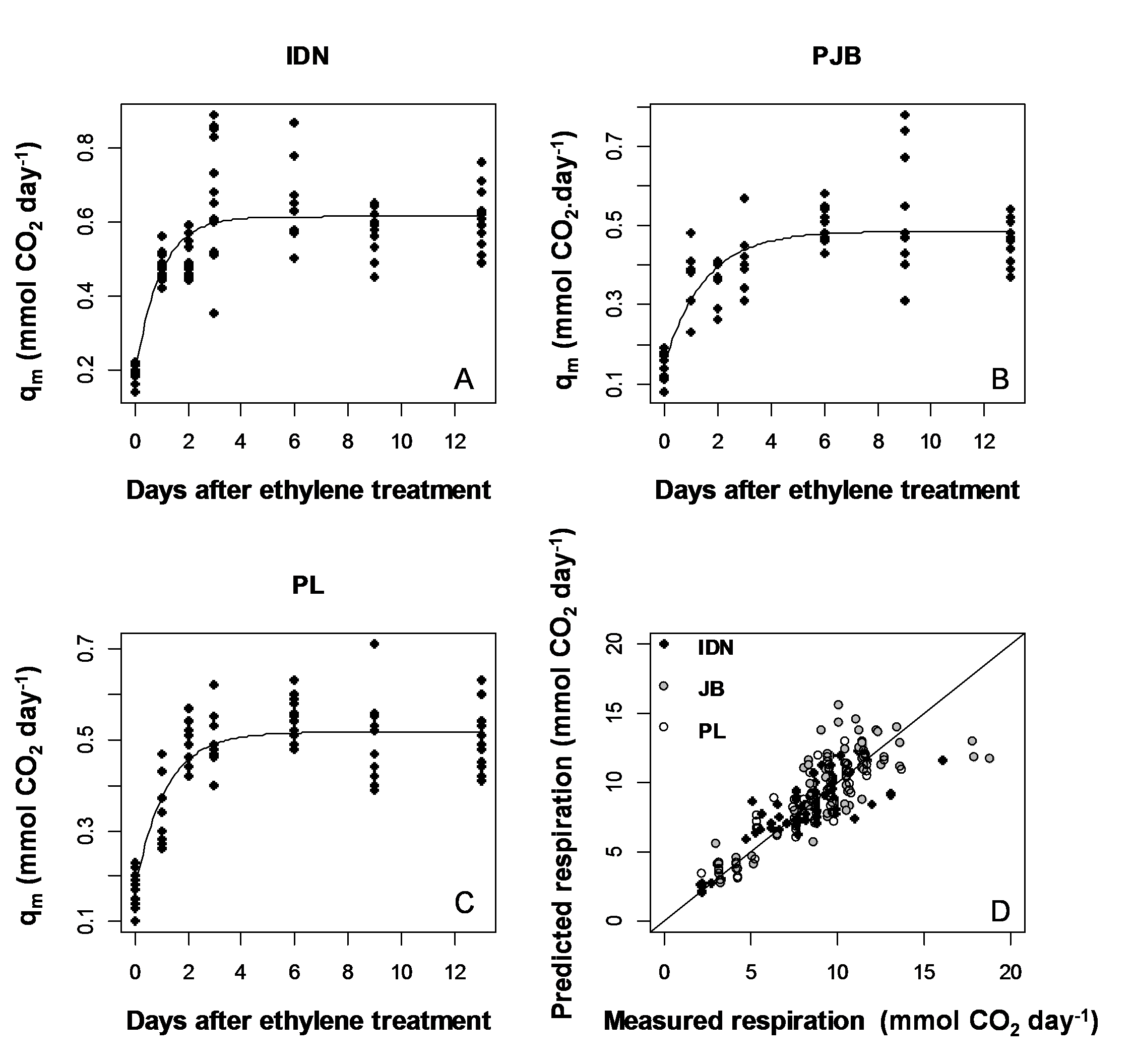

Supplement: S3 Fig — (TIF) [file pone.0126777.s004.tif]
