# Supplementary material for: A Process-Based Model of TCA Cycle Functioning to Analyze Citrate Accumulation in Pre- and Post-Harvest Fruits
Source: PLoS One. 2015 Jun 4;10(6):e0126777. doi: 10.1371/journal.pone.0126777 (PMC4456289; doi:10.1371/journal.pone.0126777)
Supplement: S1 Table — (PDF) [file pone.0126777.s005.pdf]

Table S1: description and units of parameters, constants and variables

Denomination	Description	Units
**Parameters and constants**		
C_m	Maximum absolute growth rate of pulp dry weight	g day^{-1}
$Cpyr_{cyt}$	Concentration of pyruvate in the pulp cytosolic compartment	mmol L^{-1}
$Cmal_{cyt}$	Concentration of malate in the pulp cytosolic compartment	mmol L^{-1}
DAE_{ref}	Reference day after ethylene treatment	day
f_{NH}	Fraction of N used in growth that is assimilated heterotrophically	dimensionless
$k_{i,g}$		L² day^{-1} mmol^{-1} or L day^{-1}
$K_{i,g}$		L day^{-1}
$k_{i,r}$	Parameter of the equations to calculate the rate constants and membrane permeabilities (Eq. 18-21)	L² day^{-1} mmol^{-1} or L day^{-1}
$K_{i,r}$		L day^{-1}
m_i		dimensionless
j_i		dimensionless
M_c	Molar mass of carbon	g mol^{-1}
q_{m1}		mmol CO_2 g^{-1} day^{-1}
q_{m2}	Parameters of the equation to calculate the maintenance coefficient during post-harvest ripening (Eq. 28)	dimensionless
q_{m3}		dimensionless
q_g	Growth respiration coefficient	mmol CO_2 g^{-1}
q_m	Maintenance coefficient at 20 °C	mmol CO_2 g^{-1} day^{-1}
Q_{10}	Temperature ratio of maintenance respiration	dimensionless
r_T	Added cost of translocating photosynthetates from sources to sink	%
R_m	Maximum relative growth rate of pulp dry weight	g g^{-1} day^{-1}
SDW_{ref}	Reference structural dry weight	g
t_b	x axis intercept of the linear growth phase of pulp dry weight	day
α	Concentration of carbon in glucose	dimensionless
**Variables**		
A	Ash concentration in the pulp	g g DW^{-1}
C	Carbon concentration in the pulp	g g DW^{-1}

CC	Construction cost	g glucose g^{-1}
Ccit	Citrate concentration in the pulp	mmol 100g FW^{-1}
$Ccit_{mt}$	Concentration of citrate in the pulp mitochondrial compartment	mmol L^{-1}
$Ccit_{cyt}$	Concentration of citrate in the pulp cytosolic compartment	mmol L^{-1}
$Cmal_{mt}$	Concentration of malate in the pulp mitochondrial compartment	mmol L^{-1}
$Cpyr_{mt}$	Concentration of pyruvate in the pulp mitochondrial compartment	mmol L^{-1}
DAE	Day after ethylene treatment	day
DW	Pulp dry weight	g
FW	Pulp fresh weight	g
$k_{i,g}(t)$	Rate constant during fruit growth	L^2 day^{-1} $mmol^{-1}$ or L day^{-1}
$k_{i,r}(t)$	Rate constant during fruit post-harvest ripening	L^2 day^{-1} $mmol^{-1}$ or L day^{-1}
$K_{i,g}(t)$	Membrane permeability during fruit growth	L day^{-1}
$K_{i,r}(t)$	Membrane permeability during fruit post-harvest ripening	L day^{-1}
$MCit_{t0}$	Initial citrate amount in the pulp	mmol $fruit^{-1}$
$Mcit_{mt}$	Amount of citrate in the pulp mitochondrial compartment	mmol
$Mmal_{mt}$	Amount of malate in the pulp mitochondrial compartment	mmol
$Mpyr_{mt}$	Amount of pyruvate in the pulp mitochondrial compartment	mmol
N	Nitrogen concentration in the pulp	g g DW^{-1}
Resp	Pulp respiration	mmol day^{-1}
SDW	Pulp structural dry weight	g
φ_i	Metabolic fluxes of the TCA cycle	mmol day^{-1}
θ	Air temperature	°C
