# Supplementary material for: A Process-Based Model of TCA Cycle Functioning to Analyze Citrate Accumulation in Pre- and Post-Harvest Fruits
Source: PLoS One. 2015 Jun 4;10(6):e0126777. doi: 10.1371/journal.pone.0126777 (PMC4456289; doi:10.1371/journal.pone.0126777)
Supplement: S2 Table — (PDF) [file pone.0126777.s006.pdf]

Table S2 LMM analysis of predicted and measured citrate concentration (g 100 g FW^{-1}) during fruit growth. The factors studied were fruit age, cultivar, and pruning treatment in the 2011 experiment, and fruit age, cultivar, and potassium fertilization in the 2012 experiment. Linear mixed-effects models [LMMs (Gałecki and Burzykowski, 2013)] were used to examine the relationship between citrate concentration and explanatory variables (fruit age, cultivar, treatment), and interactions. We used quadratic and cubic terms of fruit age when the curve passed through a maximum and had an asymmetrical shape. We used the lme function in the 'nlme' library (Pinheiro et al., 2013) in the statistical program R 2.14.0. "Banana plant" was treated as a random effect because banana plants were assumed to contain unobserved heterogeneity, which is impossible to model. A temporal correlation structure was used to account for temporal pseudo-replication. Model selection was made using the top-down strategy (Zuur et al., 2009): starting with a model in which the fixed component contains all the explanatory variables and interactions, we found the optimal structure of the random component. We then used the F-statistic obtained with restricted maximum likelihood (REML) estimation to find the optimal fixed structure. Finally, the significance of each factor kept in the optimal model was assessed using the F-statistic obtained with REML estimation.

F-value [a] and significance [b]			
Year	Factors [c]	Predicted citrate concentration	Measured citrate concentration
2011			
	c	20***	16***
	p	6*	Ns
	a	5649***	2703***
	a^2	224***	184***
	a^3	Ns	Ns
	p : a	22***	Ns
	c : a	27***	7***
	c : p	Ns	Ns
	c: p : a	Ns	Ns
2012			
	c	5*	28***
	f	Ns	Ns
	a	4239***	1603***
	a^2	208***	142***
	a^3	15***	Ns
	c : a	36***	8***
	c : f	Ns	Ns
	f: a	Ns	Ns
	c: f: a	Ns	Ns

[a] The F-value is given only for the factors kept in the optimal model.

[b] *** p-value < 0.001; ** p-value < 0.01; * p-value<0.05 ; Ns : not significant.

[c] Codes for factors: c=cultivar; p=pruning treatment; a=fruit age (in % of flowering-to-yellowing time); f=potassium fertilization treatment.
