# Supplementary material for: A Process-Based Model of TCA Cycle Functioning to Analyze Citrate Accumulation in Pre- and Post-Harvest Fruits
Source: PLoS One. 2015 Jun 4;10(6):e0126777. doi: 10.1371/journal.pone.0126777 (PMC4456289; doi:10.1371/journal.pone.0126777)
Supplement: S3 Table — (PDF) [file pone.0126777.s007.pdf]

Table S3 LMM analysis of predicted and measured citrate concentration (g 100 g FW^{-1}) during postharvest fruit ripening. The factors studied were ripening stage, fruit age at harvest, cultivars and pruning treatment in the 2011 experiment, and ripening stage, cultivars and potassium fertilization treatment in the 2012 experiment.

		F-value [a] and significance [b]	
Year	Factors	Predicted citrate concentration	Measured citrate concentration
2011			
	c	517***	496***
	p	Ns	Ns
	a	Ns	23***
	r	241***	21***
	r^2	36***	45***
	r^3	6*	5*
	p : r	10**	Ns
	a : c	15***	Ns
	a : r	21***	Ns
	c : r	1204***	212***
	p : a	Ns	Ns
	p : c	Ns	Ns
	a:c:r	35***	Ns
	p :a :c	Ns	Ns
	p:a:r	Ns	Ns
	p:a:c:r	Ns	Ns
2012			
	c	147***	252***
	f	Ns	Ns
	r	106***	6*
	r^2	20***	29***
	r^3	7**	Ns
	c : f	6**	Ns
	c : r	564***	104***
	f : r	Ns	Ns
	c: f : r	Ns	Ns

[a] The F-value is given only for the factors retained from the optimal model.

[b] *** p-value < 0.001; ** p-value < 0.01; * p-value<0.05 ; Ns : not significant.

[c] Codes for factors: c=cultivar; p=pruning treatment; a=fruit age at harvest; r=ripening stage; f=potassium fertilization treatment.
