# Supplementary material for: A Process-Based Model of TCA Cycle Functioning to Analyze Citrate Accumulation in Pre- and Post-Harvest Fruits
Source: PLoS One. 2015 Jun 4;10(6):e0126777. doi: 10.1371/journal.pone.0126777 (PMC4456289; doi:10.1371/journal.pone.0126777)
Supplement: S4 Table — (PDF) [file pone.0126777.s008.pdf]

Table S4 Estimated parameter values and standard errors (in parentheses) of the expolinear growth model of pulp dry weight for the three cultivars (IDN, PJB, and PL) and two contrasted fruit loads (LL: low fruit load; HL: high fruit load) in 2011.

Cultivar	Fruit load	C_m (g day^{-1})	R_m (g g^{-1} day^{-1})	t_b (day)
IDN	LL	0.34 (0.02)	0.11 (0.03)	30.1 (2.4)
IDN	HL	0.28(0.02)	0.11 (0.04)	29.7 (3.7)
JB	LL	0.54 (0.03)	0.11 (0.03)	36.1 (3.0)
JB	HL	0.40 (0.02)	0.13 (0.04)	30.4 (2.6)
PL	LL	0.36 (0.02)	0.11 (0.03)	29.9 (2.7)
PL	HL	0.32 (0.02)	0.12 (0.04)	30.92 (3.4)
