# Supplementary material for: A Process-Based Model of TCA Cycle Functioning to Analyze Citrate Accumulation in Pre- and Post-Harvest Fruits
Source: PLoS One. 2015 Jun 4;10(6):e0126777. doi: 10.1371/journal.pone.0126777 (PMC4456289; doi:10.1371/journal.pone.0126777)
Supplement: S5 Table — (PDF) [file pone.0126777.s009.pdf]

Table S5 Estimated parameter values and standard errors (in parentheses) of the expolinear growth model of pulp dry weight in the three cultivars (IDN, PJB, and PL) and the two contrasted levels of potassium fertilization (NF: no potassium fertilization; HF: high potassium fertilization) in 2012.

Cultivar	Potassium fertilization	C_m (g day^{-1})	R_m (g g^{-1} day^{-1})	t_b (day)
IDN	NF	0.29 (0.02)	0.15 (0.08)	26.5 (2.9)
IDN	HF	0.26 (0.03)	0.13 (0.08)	28.5 (5.0)
JB	NF	0.34 (0.02)	0.21 (0.14)	28.4 (2.2)
JB	HF	0.40 (0.04)	0.12 (0.07)	32.5 (5.3)
PL	NF	0.31 (0.02)	0.14 (0.06)	27.2 (3.0)
PL	HF	0.33 (0.02)	0.16 (0.10)	25.2 (3.5)
